# Comparative Safety of the BNT162b2 Messenger RNA COVID-19 Vaccine vs Other Approved Vaccines in Children Younger Than 5 Years

**DOI:** 10.1001/jamanetworkopen.2022.37140

**Published:** 2022-10-18

**Authors:** Nicole Toepfner, Wolfgang C. G. von Meißner, Christoph Strumann, Denisa Drinka, David Stuppe, Maximilian Jorczyk, Jeanne Moor, Johannes Püschel, Melanie Liss, Emilie von Poblotzki, Reinhard Berner, Matthias B. Moor, Cho-Ming Chao

**Affiliations:** 1 Department of Pediatrics, University Hospital Carl Gustav Carus, Technische Universität Dresden, Dresden, Germany; 2 Hausärzte am Spritzenhaus, Family Practice, Baiersbronn, Germany; 3 Institute of Family Medicine, University Hospital Schleswig-Holstein, Campus Luebeck, Luebeck, Germany; 4 Department of Pediatrics, University Medical Center Rostock, University of Rostock, Rostock, Germany; 5 Department of General Internal Medicine, Inselspital University Hospital Bern, Bern, Switzerland; 6 Institute of Primary Health Care (BIHAM), University of Bern, Bern, Switzerland; 7 Hausarztzentrum Greven, Family Practice, Greven, Germany; 8 Praxis für Kinder- und Jugendmedizin, Düsseldorf, Germany; 9 Praxis die Kinderaerzte, München, Deutschland; 10 Department of Nephrology and Hypertension, Inselspital University Hospital Bern, Bern, Switzerland; 11 Cardio-Pulmonary Institute, Universities of Giessen and Marburg Lung Center, member of the German Center for Lung Research, Justus Liebig University Giessen, Giessen, Germany

## Abstract

**Importance:**

SARS-CoV-2 vaccines are authorized for use in most age groups. The safety of SARS-CoV-2 vaccines is unknown in children younger than 5 years.

**Objective:**

To retrospectively evaluate the safety of the BNT162b2 vaccine used off-label in children younger than 5 years compared with the safety of non–SARS-CoV-2 vaccines in the same sample.

**Design, Setting, and Participants:**

This investigator-initiated retrospective cohort study included parents or caregivers who registered children for SARS-CoV-2 vaccination in outpatient care facilities in Germany. The study was performed as an authenticated online survey. A total of 19 000 email addresses were contacted from vaccination registration databases between April 14 and May 9, 2022. Inclusion criteria were child age younger than 5 years at the first BNT162b2 vaccination and use of a correct authentication code to prove invitation.

**Exposures:**

Off-label BNT162b2 vaccination and on-label non–SARS-CoV-2 vaccinations.

**Main Outcomes and Measures:**

Reported short-term safety data of 1 to 3 doses of 3 to 10 μg BNT162b2 in children from birth to younger than 60 months are presented. Coprimary outcomes were the frequencies of 11 categories of symptoms after vaccination with bivariate analyses and regression models adjusting for age, sex, weight, and height.

**Results:**

The study included 7806 children (median age, 3 years [IQR, 2-4 years]; 3824 [49.0%] female) who were followed up of for a mean (SD) of 91.4 (38.8) days since first BNT162b2 vaccination (survey response rate, 41.1%). A 10-μg dosage was more frequently associated with local injection-site symptoms compared with lower dosages. In the active-comparator analysis, the probability of any symptoms (odds ratio [OR], 1.62; 95% CI, 1.43-1.84), local symptoms (OR, 1.68; 95% CI, 1.38-2.05), musculoskeletal symptoms (OR, 2.55; 95% CI, 1.32-4.94), dermatologic symptoms (OR, 2.18; 95% CI, 10.7-4.45), or otolaryngologic symptoms (OR, 6.37; 95% CI, 1.50-27.09) were modestly elevated after BNT162b2 compared with non–SARS-CoV-2 vaccines, whereas the probabilities of general symptoms (OR, 0.77; 95% CI, 0.63-0.95) and fever (OR, 0.42; 95% CI, 0.32-0.55) were lower after BNT162b2. Symptoms requiring hospitalization (n = 10) were reported only at BNT162b2 dosages above 3 μg.

**Conclusions and Relevance:**

In this cohort study, the symptoms reported after BNT162b2 administration were comparable overall to those for on-label non–SARS-CoV-2 vaccines in this cohort of children younger than 5 years. The present data may be used together with prospective licensure studies of BNT162b2 efficacy and safety and could help guide expert recommendations about BNT162b2 vaccinations in this age group.

## Key Points

**Question:**

Is the BNT162b2 SARS-CoV-2 vaccine safe in children younger than 5 years?

**Findings:**

In this cohort study based on a survey of guardian-reported safety profiles of BNT162b2 in 7806 children, higher dosages of BNT162b2 were significantly associated with injection-site reactions. Compared with approved non–SARS-CoV-2 vaccines, BNT162b2 was associated with significantly more frequent injection-site, musculoskeletal, dermatologic, or otolaryngologic symptoms but fewer general symptoms and fever after vaccination.

**Meaning:**

In this study, the overall frequency of adverse events after vaccination with BNT162b2 was comparable with the frequency of adverse events after vaccination with approved non–SARS-CoV-2 vaccines in children younger than 5 years.

## Teaser

This cohort study compares the safety of off-label use of the BNT162b2 vaccine with non–SARS-CoV-2 vaccines in children younger than 5 years.

## Introduction

The safety and efficacy of the SARS-CoV-2 messenger RNA (mRNA) vaccine BNT162b2 has been widely studied in children and adolescents 5 years or older.^1,2,3,4,5^ However, in children younger than 5 years, the SARS-CoV-2 vaccine BNT162b2 only recently received an emergency use approval by the US Food and Drug Administration, whereas decisions of other major regulatory agencies are pending.^6,7^ Public news media have reported since the middle of 2021 that German initiatives by laypeople or medical care professionals have organized off-label SARS-CoV-2 vaccinations,^8,9,10,11^ including children who themselves or whose family members have comorbidities or medications that may increase their risk for severe COVID-19. Off-label administration of SARS-CoV-2 vaccines to children younger than 5 years is permitted according to German law after obtaining written informed consent but remains at parents’, legal guardians’, or health care professionals’ risk or liability.^12,13^

Parents or legal guardians may seek opportunities to vaccinate children against SARS-CoV-2 to obtain, according to their individual estimation, the best attainable protection against COVID-19 for their child and/or potentially diminish the risk of postacute syndromes or virus transmission. On February 17, 2022, the German government’s COVID-19 advisory council stated that children’s welfare in times of pandemic requires prioritization, including systematic efforts to study potential adverse effects of SARS-CoV-2 vaccinations.^14,15^ No safety data currently are available for BNT162b2 in children younger than 5 years. This study retrospectively evaluates the safety of the BNT162b2 vaccine used off-label in children younger than 5 years compared with the safety of non–SARS-CoV-2 vaccines in the same sample.

## Methods

### Study Design

The Safety of the BNT162b2 mRNA COVID-19 Vaccine in Children below 5 Years in Germany (CoVacU5) study was designed as a retrospective cohort study to evaluate the safety of the BNT162b2 mRNA vaccine in children vaccinated before completing the fifth year of age. Inclusion criteria were being registered in a database for at least 1 dose of BNT162b2 that was administered before reaching the age of 5 years and having written informed consent from a parent or legal representative to participate in the current study. Parents or legal representatives also gave written individual informed consent to the vaccinations and were informed by the attending physicians about potential adverse effects and liability of this off-label medicine use under German law. The attending physicians were themselves required by the German Infection Protection Act and by their professional status to report all unexpected or severe adverse effects experienced by the children to the German Federal Institute for Drugs and Medical Devices independently from the current study. Eligible participants were identified from electronic registration databases from 2 nationwide layperson-initiated SARS-CoV-2 vaccination social media programs (U12Schutz and Bildung Aber Sicher) in Germany that helped to establish contact between the study team and 21 outpatient care facilities where off-label vaccinations with BNT162b2 were administered. Exclusion criteria were duplicate responses overlapping in all of the following: age, sex, weight, height, and authentication code, without specification that children were twins or triplets. Respondents who lacked an authentication code as proof of invitation were also excluded. In addition, BNT162b2 vaccinations reported to have occurred before May 2021 and vaccinations with SARS-CoV-2 vaccines other than BNT162b2 were excluded from analysis. Of note, the vaccines were neither administered as part of the study nor was the study planned or initiated before vaccination, and no medical records data were accessed for this study. The current study protocol was approved by the Ethics Committee of the University of Rostock, Rostock, Germany. The study was prospectively registered in the German Clinical Trials Register^16^ and was conducted with adherence to the Declaration of Helsinki.^17^ This study follows the Strengthening the Reporting of Observational Studies in Epidemiology (STROBE) reporting guideline for observational studies.

Within the study period of April 14 to May 9, 2022, the parents or caregivers were contacted twice via email addresses registered in the databases and were invited to participate in a web-based survey. All invitees received an 8-digit authentication code to ensure participation of only those who had effectively registered at least 1 child for vaccination. On May 9, 2022, the study database was closed, and data were extracted for analysis in a fully anonymous manner.

### Study Procedure

All study data were collected and managed using REDCap^18^ (Research Electronic Data Capture) tools hosted at the University Medical Center Dresden, Dresden, Germany. The survey was designed to be completed within approximately 5 minutes if no symptoms were reported, and it was pretested in 20 pilot test runs. An introductory page revealed the study purpose and obtained written informed consent for voluntary participation and allowing data use for research purposes. Demographic information, comorbidities, and medications of the children were subsequently collected, and precise symptoms, including their timing after vaccination, were determined by close-ended questions and free-text responses. The symptom categories were injection-site symptoms; general reactions; fever; skin changes; any symptoms of the musculoskeletal, cardiovascular, pulmonary, or nervous system; gastrointestinal tract symptoms; any symptoms of the ear, neck, or throat; psychological symptoms; or susceptibility for infections. For individual symptoms and categories, radio button choice questions were used, and for the “any symptoms” analysis, both free-text responses and radio button choice questions were included. In addition, free-text responses were queried for the German terms for *myocarditis*, *pericarditis*, and *hospitalization*. Furthermore, the occurrence of missing days from a childcare institution or school was recorded. A 10-point Likert scale was used to determine whether the reported postvaccination symptoms in the children were considered to be threatening, as used previously.^19^ For comparison in an active-comparator analysis, information was simultaneously collected regarding postvaccination symptom categories of any other non–SARS-CoV-2 vaccination administered in the same children since January 15, 2022.

### Outcomes

The primary outcome of the current study was the probability of self-reported categorized symptoms after BNT162b2 vaccination compared with vaccination with non–SARS-CoV-2 vaccines with particular respect to serious adverse events (SAEs), the frequencies of categorized symptoms occurring after BNT162b2 vaccine administration with stratification by age group and dosage, and the general susceptibility to infections and institutional missing days. Secondary outcomes were the total number, duration, and consequences of symptoms occurring after vaccination with BNT162b2.

### Statistical Analysis

All eligible participants were enrolled without sample size calculations. Statistical analyses were performed using MATLAB software, version R2020a (MathWorks Inc) and Stata software, version 15 (StataCorp LLC) according to an investigational plan (eAppendixes 1 and 2 in the Supplement). A χ^2^ or Fisher exact test was used to compare categorical variables over predefined strata of age groups and BNT162b2 dosage. The unpaired, 2-tailed *t* test or Wilcoxon rank sum test was used for comparisons of continuous variables as appropriate. Multivariable logistic models were performed with any symptom or the 11 individual categories of postvaccination symptoms as dependent variables, with BNT162b2 vs non–SARS-CoV-2 vaccines as the variable of interest. Duration and impact of the symptoms were assessed in additional logistic models. In addition, multivariate negative binomial regression was used for the number of symptoms as the dependent variable. All regression models were adjusted for age, sex, weight, and height. For bivariate analyses and regression models, *P* values and 95% CIs were adjusted for multiple testing of 11 symptom categories using the Bonferroni method. An adjusted 2-tailed *P* < .05 (signifying an unadjusted *P* < .004) was considered significant. A robustness check of the data was performed by multiple imputation (eAppendix 2 in the Supplement). Complete variables serving as explanatory variables were included in the imputation models. Additional, aggregated geolocation dummy variables based on postal code were used as regression variables in the imputation models if they obtained complete data. In a prespecified sensitivity analysis, participants without a verifiable^20^ lot number of BNT162b2 vaccinations were excluded. Additional sensitivity analyses that included strata with or without comorbidities, the entire data set without time restriction, or geolocation or vaccinating clinic as a random variable in mixed-effects models were performed.

## Results

### Study Population

**Figure 1.**
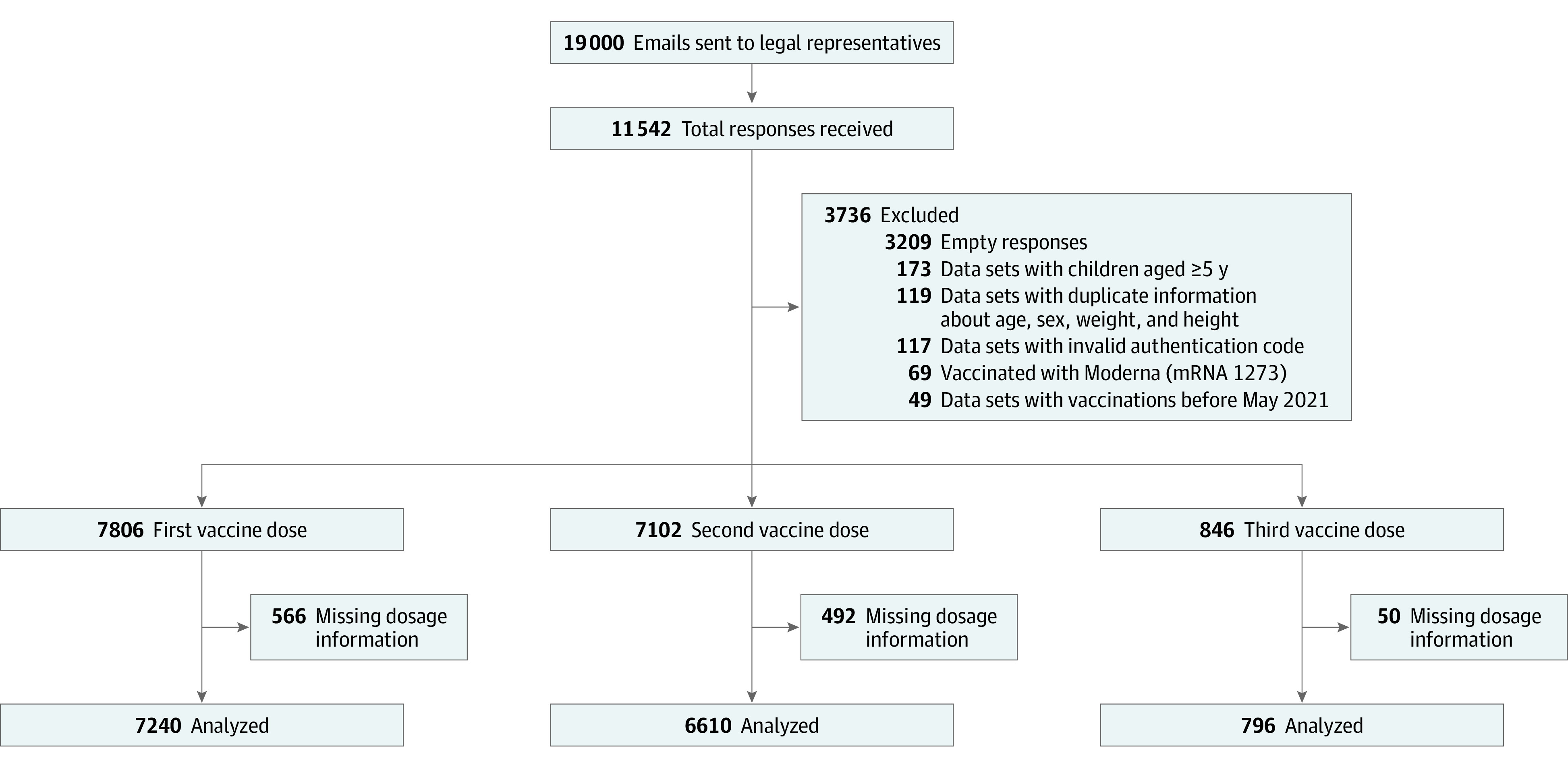
Overview of Eligible and Enrolled Study Participants The number of participants contacted via email, all excluded responses, and the final number of BNT162b2 vaccine administrations analyzed are shown.

The study included 7806 children (median age, 3 years [IQR, 2-4 years]; 3824 girls [49.0%] and 3977 boys [51.0%]), representing a response rate of 41.1% of 19 000 eligible individuals who were contacted. We included 338 children younger than 12 months, 1272 children aged 12 to younger than 24 months, and 5629 children aged 24 to younger than 60 months at the time of their first BNT162b2 vaccination. Some children were older than 5 years at their second or third BNT162b2 vaccination. Figure 1 shows the flowchart of participant recruitment, numbers of excluded survey entries, and reasons for exclusions. The current analysis included 7806 children administered at least 1 dose of BNT162b2 with a mean (SD) follow-up of 91.4 (38.8) days since the first dose of BNT162b2, at least 2 doses in 7102 children, and 3 doses in 846 children. For 7750 of the 15 754 vaccinations with BNT162b2 (49.2%), manufacturer-verified lot numbers were reported. Population characteristics, including dosages and intervals between vaccines, are given in eTable 1 in the Supplement. Among 684 of 7806 responding participants (8.8%) with comorbidities (eTable 1 in the Supplement), the most common diseases were pulmonary (190 [2.4%]) or cardiovascular diseases (90 [2.1%]) and chromosomal aberrations (166 [1.2%]) (eTable 2 in the Supplement).

### Frequency of Postvaccination Symptoms After BNT162b2 Vaccination

**Figure 2.**
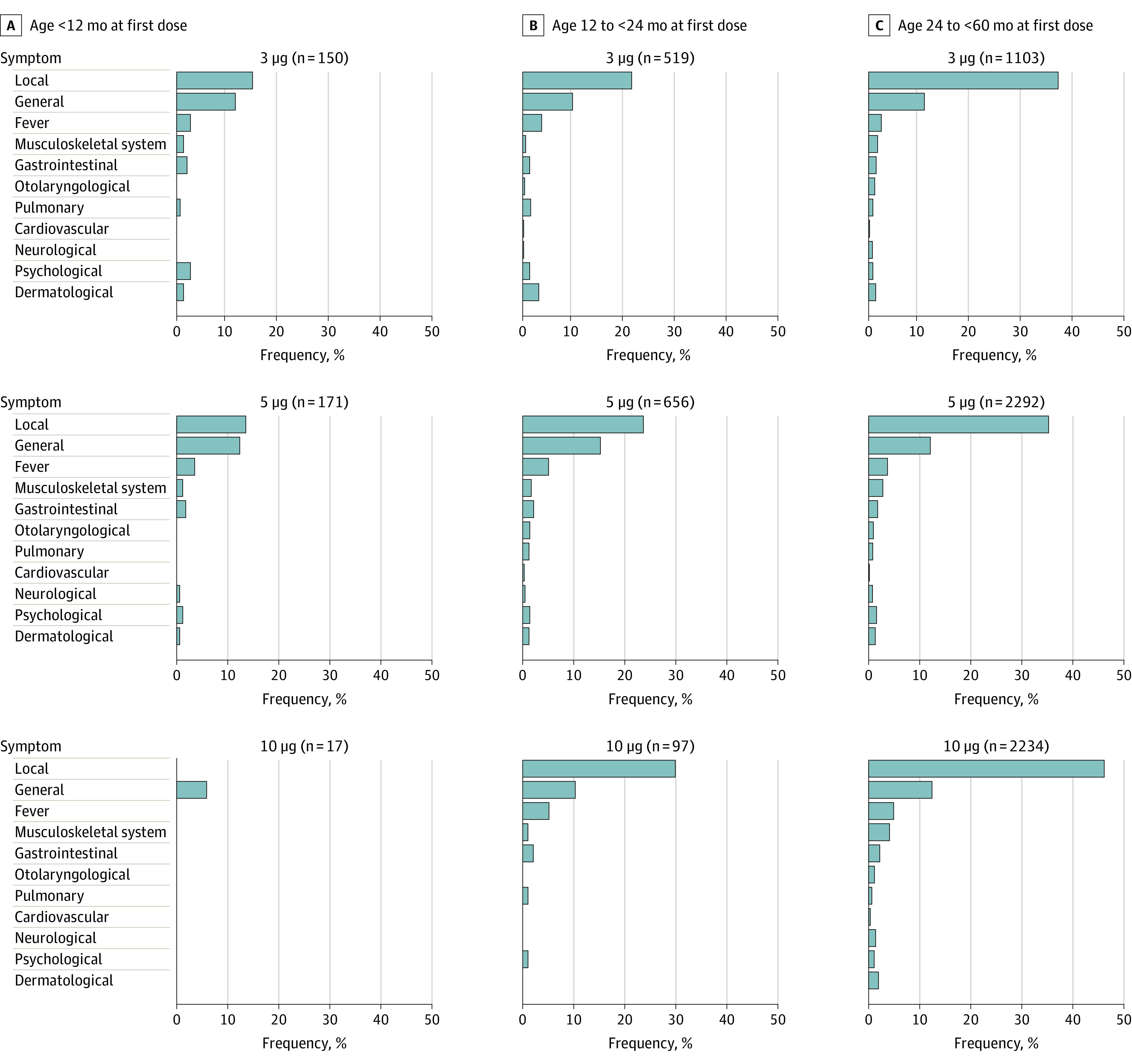
Symptoms Reported After the First Dose of BNT162b2 Vaccine According to Age Group

**Figure 3.**
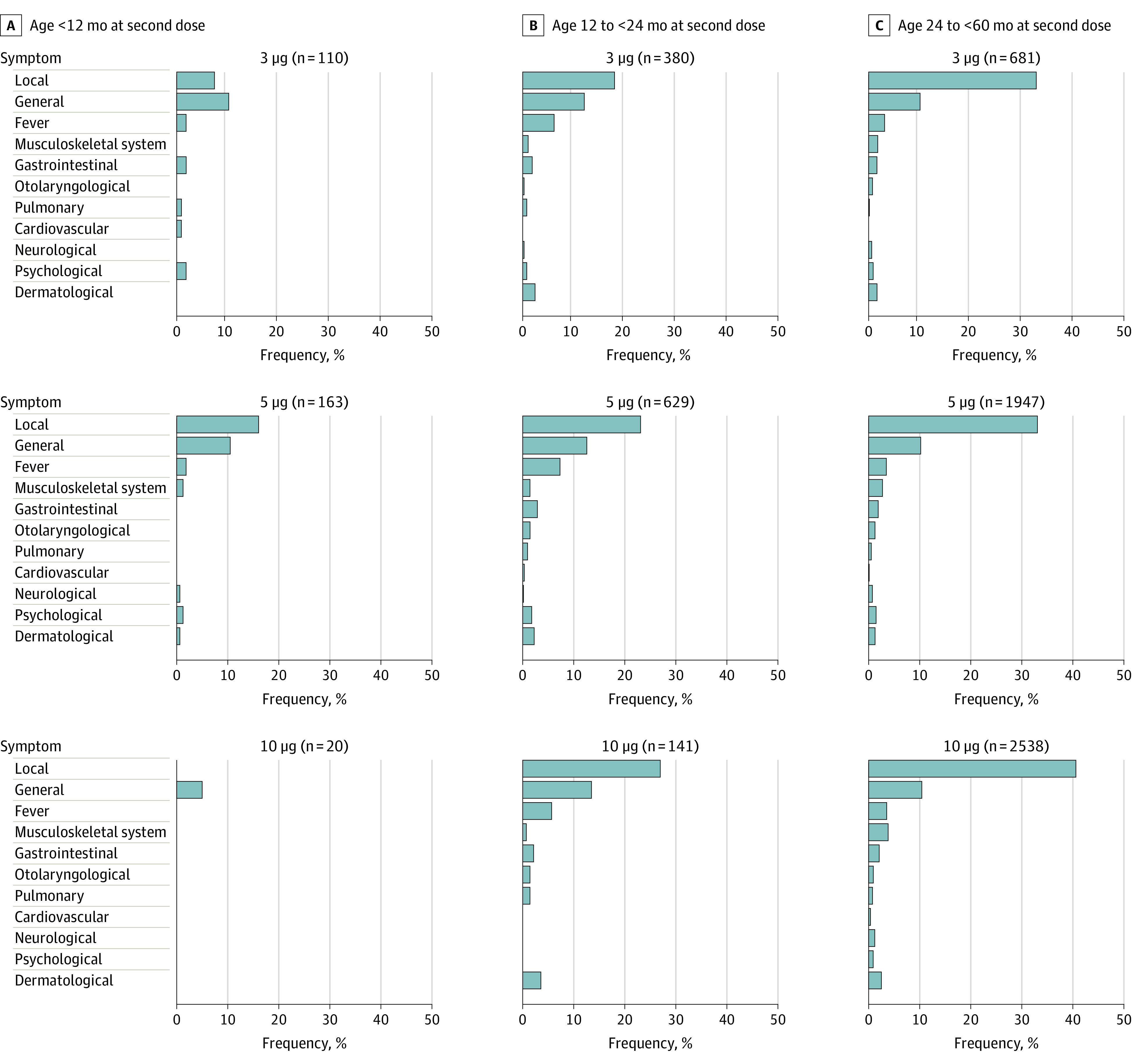
Symptoms Reported After the Second Dose of BNT162b2 Vaccine According to Age Group

A descriptive analysis of postvaccination symptoms was performed. Frequencies of categorized symptoms occurring after the first, second, or third BNT162b2 vaccination were stratified for age groups and BNT162b2 vaccine dosages (Figure 2 and Figure 3; eTables 3, 4, and 5 in the Supplement). Although most categories were comparable across the strata, local injection-site symptoms were more frequently reported for those older than 24 but younger than 60 months than for younger children (eTables 3 and 4 in the Supplement). No age-dependent effects of reported symptoms or dose-dependent effects except injection-site symptoms were found in children aged 24 to younger than 60 months. Most BNT162b2 postvaccinations symptoms were judged to be of no or minimal subjective severity and did not lead to any missing days in school or childcare (eTable 6 in the Supplement). We retrospectively defined postvaccination mortality (0 of 7806 [0%]) and symptoms requiring inpatient treatment (10 of 7806 [0.1%]) as SAEs. We additionally considered symptoms as of concern when they lasted more than 3 months (2 of 7806 respondents [.03%]) and of unknown significance when they were currently ongoing (40 of 7806 [0.5%]). Symptoms of the 10 children with SAEs lasted a mean (SD) of 12.2 (18.6) days (maximum, 60 days) and were observed after BNT162b2 vaccination at 5 μg, 10 μg, or unknown/other dosage; no SAE symptoms were observed after the 3-μg dosage (Table 1) or after the third vaccination with BNT162b2. A full analysis of postvaccination symptoms, including time of onset, duration, and self-perceived severity, is given for all individual symptom categories in eTables 7 through 16 in the Supplement. Of note, no respondents indicated that a child had received a diagnosis of myocarditis.

**Table 1.** Characteristics of the Children Younger Than 5 Years With Reported Serious Adverse Events After BNT162b2 Vaccine Administration^a^

Characteristic	Overall (N = 10)	BNT162b2 vaccination	
		First (n = 10)	Second (n = 9)
Sex			
Female	7 (70.0)	NA	NA
Male	3 (30.0)	NA	NA
Age, median (IQR), y	4 (1.0)	NA	NA
Comorbidities (yes)	6 (60.0)	NA	NA
Long-term medication	2 (20.0)	NA	NA
Inpatient treatment	10 (100.0)	7 (70.0)	3 (30.0)
Dosage, μg			
3	NA	0	0
5	NA	3 (30.0)	4 (40.0)
10	NA	5 (50.0)	5 (50.0)
Unknown	NA	2 (20.0)	1 (10.0)
Symptom duration			
Mean (SD) [maximum], d	12.2 (18.6) [60]	4.3 (5.4) [14]	9.3 (20.8) [60]
>90 d	0	1 (10.0)	0
Ongoing	1 (10.0)	0	0
Symptoms			
Local	5 (50.0)	5 (50.0)	3 (33.3)
General	5 (50.0)	3 (30.0)	3 (33.3)
Fever	2 (20.0)	2 (20.0)	0
Musculoskeletal system	4 (40.0)	2 (20.0)	3 (33.3)
Gastrointestinal	1 (10.0)	0	1 (11.1)
Otolaryngological	3 (33.3)	2 (22.2)	1 (12.5)
Pulmonary	4 (40.0)	4 (40.0)	0
Cardiovascular	4 (40.0)	2 (20.0)	3 (33.3)
Neurologic	3 (30.0)	1 (10.0)	2 (22.2)
Psychological	1 (10.0)	0	1 (11.1)
Dermatologic	2 (20.0)	1 (10.0)	1 (11.1)

Abbreviation: NA, not applicable.

^a^ Data are presented as number/total number (%) of study participants unless otherwise indicated.

### Active Comparator Analysis of BNT162b2 vs Non-BNT162b2 Vaccinations

A comparison of the symptoms that occurred after off-label vaccination with BNT162b2 with those occurring after on-label vaccinations against pathogens other than SARS-CoV-2 was performed within the same group of participants (eTable 17 in the Supplement). The symptoms of all 4570 individuals receiving BNT162b2 vaccination and the 1491 receiving non–SARS-CoV-2 vaccinations between January 15, 2022, and May 9, 2022, were assessed to minimize potential differences in recall bias between vaccine groups. Any symptoms were observed after 2323 of 4570 BNT162b2 vaccinations (50.8%) and 564 of 1491 non–SARS-CoV-2 vaccinations (37.8%). In logistic regression models adjusted for age, sex, weight, and height, the probability of any symptoms (odds ratio [OR], 1.62; 95% CI, 1.43-1.84), local injection-site symptoms (OR, 1.68; 95% CI, 1.38-2.05), musculoskeletal symptoms (OR, 2.55; 95% CI, 1.32-4.94), dermatologic symptoms (OR, 2.18; 95% CI, 10.7-4.45), or otolaryngological symptoms (OR, 6.37; 95% CI, 1.50-27.09) were modestly elevated after BNT16in2b2 compared with non–SARS-CoV-2 vaccines (Table 2). However, the probabilities of general symptoms (OR, 0.77; 95% CI, 0.63-0.95) or fever (OR, 0.42; 95% CI, 0.32-0.55) were lower after BNT162b2 vaccinations than after non-SARS-CoV-2 vaccination. The probabilities of all other symptom categories were comparable between BNT162b2 and non–SARS-CoV-2 vaccinations (Table 2). A symptom duration of longer than 3 months was reported after 1 of 4570 BNT162b2 vaccinations (0.02%) and none of the non–SARS-CoV-2 vaccinations. Symptoms were currently ongoing after 24 of 4570 BNT162b2 vaccinations (0.5%) and 1 of 1491 non–SARS-CoV-2 vaccinations (0.1%). As in the entire data set, 2 of 7806 individuals (0.03%), symptoms lasted longer than 90 days, and in 10 of 7806 children (0.1%), hospitalizations were reported for SAEs. Symptoms requiring inpatient treatment were reported after 5 of 4570 BNT162b2 vaccinations (0.1%) and none of the non–SARS-CoV-2 vaccinations.

**Table 2.** Active-Comparator Analysis of Symptoms Occurring After BNT162b2 and Non-BNT162b2 Vaccinations

Variable	Frequencies, No./total No. (%)		Logistic regression	
	BNT162b2	Non-BNT162b2	OR (95% CI)	*P* value
Disposition				
Physical rest	706/4570 (15.4)	261/1491 (17.5)	0.89 (0.76-1.05)	.16
Antipyretics	339/4570 (7.4)	220/1491 (14.8)	0.54 (0.45-0.66)	<.001
Ambulatory	41/4570 (0.9)	7/1491 (0.5)	1.78 (0.78-4.04)	.17
Inpatient	5/4570 (0.1)	0/1491 (0.0)	NA	NA
Mortality	0/4570 (0.0)	0/1491 (0.0)	NA	NA
Symptoms				
Duration >90 d	1/4570 (0.0)	0/1491 (0.0)	NA	NA
Duration ongoing	24/4570 (0.5)	1/1491 (0.1)	NA	NA
Any symptom reported	2323/4570 (50.8)	564/1491 (37.8)	1.62 (1.43-1.84)	<.001
Local	1808/4520 (40.0)	380/1491 (25.5)	1.68 (1.38-2.05)^a^	<.001^a^
General	874/4506 (19.4)	388/1491 (26.0)	0.77 (0.63-0.95)^a^	.005^a^
Fever	305/4570 (6.7)	256/1491 (17.2)	0.42 (0.32-0.55)^a^	<.001^a^
Musculoskeletal system	193/4496 (4.3)	21/1491 (1.4)	2.55 (1.32-4.94)^a^	<.001^a^
Gastrointestinal	151/4493 (3.4)	36/1491 (2.4)	1.54 (0.89-2.65)^a^	.28^a^
Otolaryngologic	84/4477 (1.9)	4/1491 (0.3)	6.37 (1.50-27.09)^a^	.004^a^
Pulmonary	64/4477 (1.4)	7/1491 (0.5)	2.93 (0.94-9.13)^a^	.09^a^
Cardiovascular	17/4481 (0.4)	4/1491 (0.3)	1.36 (0.28-6.69)^a^	>.99^a^
Neurologic	64/4470 (1.4)	7/1491 (0.5)	2.52 (0.81-7.85)^a^	.24^a^
Psychological	87/4468 (1.9)	15/1491 (1.0)	2.29 (1.00-5.25)^a^	.06^a^
Dermatologic	128/4456 (2.9)	19/1491 (1.3)	2.18 (1.07-4.45)^a^	.02^a^

Abbreviations: NA, not applicable; OR, odds ratio.

^a^ Adjusted for multiple testing by Bonferroni correction. Analysis restricted to vaccines administered from January 15 to May 9, 2022. All individuals in the non-BNT162b2 group had also received at least 1 dose of BNT162b2 between May 1, 2021, and May 9, 2022, but here the symptoms occurring after the non-BNT162b2 are reported.

### Sensitivity Analyses

The current data set contained a number of less stringently verifiable participants who entered no lot number of the BNT162b2 vaccine. Thus, we performed a prespecified sensitivity analysis and reanalyzed the data presented in eTables 3 through 5 in the Supplement. Similarly, multiple imputations of missing data (eTable 18 in the Supplement) followed by reanalysis of the data are presented in eTables 3 through 5 in the Supplement. These new analyses yielded no differences in results. In further sensitivity analyses, we adjusted the models for participants’ geolocation or vaccinating clinic, analyzed strata with and without comorbidities, or included the entire study period instead of the restriction to January 15 to May 9, 2022, but the results were not affected.

## Discussion

The CoVacU5 study is an industry-independent retrospective cohort study designed to assess the self-reported short-term safety of BNT162b2 vaccines in young children in a setting where parents or caregivers had decided beforehand and independently from this study to seek off-label vaccination for their children. This study included the BNT162b2 vaccination experience of 7806 children. Because of the large study population and the different dosages investigated, CoVacU5 expands the available knowledge of mRNA vaccination in children younger than 5 years in addition to the results of the ongoing industry-sponsored BNT162b2 phase 1 to 3 studies conducted in this age group.^21^

This study revealed a similar overall safety when compared with existing non–SARS-CoV-2 vaccines administered in the same study population, with minor differences in the probabilities of injection-site symptoms, fever, musculoskeletal symptoms, or otolaryngologic symptoms compared with non–SARS-CoV-2 vaccinations. An increased frequency of injection-site symptoms was detected in children older than 24 to younger than 60 months who were administered 10 μg of BNT162b2, which should be taken into consideration in future dosage-finding strategies in this age group and be carefully weighed against potential improvements in immunogenicity at higher dosage. In 40 of 7806 individuals (0.5%), symptoms were currently ongoing and thus of unknown significance. In 2 of 7806 individuals (0.03%), symptoms lasted longer than 90 days, and in 10 of 7806 children (0.1%), hospitalizations were reported for SAEs. Although the circumstances of these SAEs are not precisely documented, no SAEs were reported for children administered the low dosage of 3 μg, which is currently evaluated in the BNT162b2 licensure study.^21^ In addition, no mortality was reported.

A relevant support for the reliability of our study result is that all physicians who administer vaccines in Germany are obliged by federal law to report all severe or unexpected vaccine-related adverse effects to the Federal Institute for Vaccines and Biomedicines (Paul-Ehrlich Institute). In addition to the data presented in this study, the Paul-Ehrlich Institute had not received any reports of unexpected or severe adverse reactions of BNT162b2 in children younger than 5 years as of March 31, 2022.^22^

### Limitations

The current study has some limitations. First, this study relies on retrospective self-reported data by parental recall, which may not directly reflect what a child had experienced and contains a risk of recall bias (ie, not recalling some non-life-threatening symptoms several months later). The low response rate of 41.1% is a potential source of bias, possibly because the generic survey invitation email could not contain details about the topic for data protection reasons. However, the extensive public debate about SARS-CoV-2 vaccinations (including their potential adverse effects), the off-label use investigated, and the study results showing dosage-dependent associations between vaccination and local reactions in children older than 24 to younger than 60 months suggest that symptoms were adequately reported. In addition, the retrospective design did not allow for a structured real-time documentation of symptoms or an estimation of causality and severity to infer the frequency of vaccine-related SAEs, but such data should be expected from the ongoing prospective, manufacturer-sponsored BNT162b2 studies.^21^ Because vaccinations themselves were not directly part of the study, the study relied on vaccination information as reported by the respondents. However, a potential risk of manipulation by individual respondents was minimized by dispensing study participation codes only via vaccinating centers or initiatives and the availability of BNT162b2 lot numbers. Moreover, potential duplicate entries were identified by overlap in demographic data and were removed.

The reported postvaccination symptoms could potentially be unrelated to the vaccine. To mitigate this problem, a non-BNT162b2 vaccination group as an active comparator was included that yielded an overall similar probability of postvaccination symptoms for most symptom categories within the last 3 months. In the time frame of this analysis, vaccinations administered from January 15 to May 9, 2022, concurred with the peak of the Omicron BA.1 and BA.2 waves, potentially leading to some symptoms being caused by undiagnosed SARS-CoV-2 but not any of the vaccines. These waves would have affected both analyzed groups similarly and do not likely bias the observed findings.

Finally, the vaccines administered in the current population were used off-label. Off-label use is common and constitutes up to 25% of all medications in pediatric care,^23^ but administration of a new vaccine in off-label use constitutes an even more particular situation. There was extensive public discussion on SARS-CoV-2 vaccination of children in Germany even in age groups in which the vaccine had already been licensed. However, because it was publicly known that a large group of young children under 5 years in Germany was being vaccinated off-label, it appeared to be important from both scientific and ethical points of view to collect all available information, analyze these data in a scientifically sound matter, and make this information publicly available. Of course, this was not a prospective, randomized, and controlled study design, and a residual risk of bias remains. Nevertheless, the scientific use and thorough evaluation of existing, retrospectively self-reported safety data for SARS-CoV-2 vaccines in off-label use appeared mandatory and may, respecting all given limitations, expand the scientific knowledge based on real-world medical practice.

## Conclusion

The data from this cohort study provide evidence for a self-reported safety profile of the BNT162b2 vaccine that is comparable to non–SARS-CoV-2 vaccines in this large cohort of children younger than 5 years. These data may be helpful in safety considerations for individual decision-making and may add to data expected from prospective licensure studies for expert recommendations about BNT162b2 vaccinations in this age group, even after the completion of the ongoing phase 1/2/3 study of BNT162b2.^21^

## Notes

Supplement. **eAppendix 1.** Statistical Analysis Plan **eAppendix 2.** Supplementary Methods **eTable 1.** Characteristics of Children Younger Than 5 Years Who Received the BNT162b2 Vaccination **eTable 2.** Comorbidities, n (%) **eTable 3.** Post-vaccination Symptoms After First BNT162b2 Vaccine Dose, n/N (%) **eTable 4.** Post-vaccination Symptoms After Second BNT162b2 Vaccine Dose, n/N (%) **eTable 5.** Post-vaccination Symptoms After Third BNT162b2 Vaccine Dose, n/N (%) **eTable 6.** Post-vaccination Symptoms After Any BNT162b2 Vaccine Dose, n/N (%) **eTable 7.** Local Symptoms **eTable 8.** General Symptoms **eTable 9.** Musculoskeletal Symptoms **eTable 10.** Gastrointestinal Symptoms **eTable 11.** Otolaryngological Symptoms **eTable 12.** Pulmonary Symptoms **eTable 13.** Cardiovascular Symptoms **eTable 14.** Neurological Symptoms **eTable 15.** Psychological Symptoms **eTable 16.** Dermatological Symptoms **eTable 17.** Non-BNT162b2 Vaccines Since Jan. 15^th^ 2022, n (%) **eTable 18.** Counts of Missing Data, n/N(%)
