# Supplementary material for: Comparative Safety of the BNT162b2 Messenger RNA COVID-19 Vaccine vs Other Approved Vaccines in Children Younger Than 5 Years
Source: JAMA Netw Open. 2022 Oct 18;5(10):e2237140. doi: 10.1001/jamanetworkopen.2022.37140 (PMC9579908; doi:10.1001/jamanetworkopen.2022.37140)
Supplement: Supplement. — eAppendix 1. Statistical Analysis Plan eAppendix 2. Supplementary Methods eTable 1. Characteristics of Children Younger Than 5 Years Who Received the BNT162b2 Vaccination eTable 2. Comorbidities, n (%) eTable 3. Post-vaccination Symptoms After First BNT162b2 Vaccine Dose, n/N (%) eTable 4. Post-vaccination Symptoms After Second BNT162b2 Vaccine Dose, n/N (%) eTable 5. Post-vaccination Symptoms After Third BNT162b2 Vaccine Dose, n/N (%) eTable 6. Post-vaccination Symptoms After Any BNT162b2 Vaccine Dose, n/N (%) eTable 7. Local Symptoms eTable 8. General Symptoms eTable 9. Musculoskeletal Symptoms eTable 10. Gastrointestinal Symptoms eTable 11. Otolaryngological Symptoms eTable 12. Pulmonary Symptoms eTable 13. Cardiovascular Symptoms eTable 14. Neurological Symptoms eTable 15. Psychological Symptoms eTable 16. Dermatological Symptoms eTable 17. Non-BNT162b2 Vaccines Since Jan. 15th 2022, n (%) eTable 18. Counts of Missing Data, n/N(%) [file jamanetwopen-e2237140-s001.pdf]

Supplementary Online Content

Toepfner N, von Meißner WCG, Strumann C, et al. Comparative safety of the BNT162b2 messenger RNA COVID-19 vaccine vs other approved vaccines in children younger than 5 years. *JAMA Netw Open*. 2022;5(10):e2237140. doi:10.1001/jamanetworkopen.2022.37140

eAppendix 1. Satistical Analysis Plan
eAppendix 2. Supplementary Methods
eTable 1. Characteristics of Children Younger Than 5 Years Who Received the BNT162b2 Vaccination
eTable 2. Comorbidities, n (%)
eTable 3. Post-vaccination Symptoms After First BNT162b2 Vaccine Dose, n/N (%)
eTable 4. Post-vaccination Symptoms After Second BNT162b2 Vaccine Dose, n/N (%)
eTable 5. Post-vaccination Symptoms After Third BNT162b2 Vaccine Dose, n/N (%)
eTable 6. Post-vaccination Symptoms After Any BNT162b2 Vaccine Dose, n/N (%)
eTable 7. Local Symptoms
eTable 8. General Symptoms
eTable 9. Musculoskeletal Symptoms
eTable 10. Gastrointestinal Symptoms
eTable 11. Otolaryngological Symptoms
eTable 12. Pulmonary Symptoms
eTable 13. Cardiovascular Symptoms
eTable 14. Neurological Symptoms
eTable 15. Psychological Symptoms
eTable 16. Dermatological Symptoms
eTable 17. Non-BNT162b2 Vaccines Since Jan. 15th 2022, n (%)
eTable 18. Counts of Missing Data, n/N (%)

This supplementary material has been provided by the authors to give readers additional information about their work.

© 2022 Toepfner N et al. *JAMA Network Open.*

Statistical analysis plan		
Section 1: Administrative information		
Title and study registration	1a	Safety of vaccination (Comirnaty, BNT162b2) against COVID-19 in Children age < 5 years in Germany (CoVacU5)
	1b	DRKS-ID: DRKS00028759
SAP version	2	SAP version V3.0
Protocol version	3	3.0
SAP revisions	4a	SAP revision history:<br>V2.0: May 4th 2022<br>Adaptation in effective survey duration and database freezing time point for practical reasons (inclusion of the weekend of April 30th/May 2nd 2022 in the survey), decision not based on interim analyses.<br><br>V3.0: May 11th 2022<br>Adaptation in effective survey duration and database freezing time point for practical reasons (inclusion of another week until May 9th 2022 in the survey), decision not based on interim analyses.<br>Adjustment for multiplicity made.<br>Age groups changed to include also 0-6 months olds.
	4b	Justification for each SAP revision:<br>V2.0:<br>Adaptation in effective survey duration and database freezing time point for practical reasons (inclusion of the weekend of April 30th/May 2nd 2022 in the survey), decision not based on interim analyses.<br><br>V3.0:<br>Adaptation in effective survey duration and database freezing time point for practical reasons (inclusion of another week until May 9th 2022 in the survey), decision not based on interim analyses.<br>Adjustment for multiplicity was mandated as per journal requirements.<br>Age groups: Better conforms to inclusion criteria to assess all participants aged <5 years.
	4c	Timing of SAP revisions in relation to planned repetitive analyses: No repetitive analyses planned, primary analysis had not started affected when the change was made to prolong the study until May 9th 2022.
Roles and responsibility	5	Names, affiliations, and roles of SAP contributors:<br><br>Matthias B. Moor, Department of Nephrology and Hypertension, University Hospital Bern, Bern, Switzerland<br>Christoph Strumann, Institute of Family Medicine, University Hospital Schleswig-Holstein, Campus Luebeck, Luebeck, Germany<br>Cho-Ming Chao, University Children's Hospital, University Medical Center Rostock, Rostock, Germany.<br>Nicole Toepfner, Department of Pediatrics, Carl Gustav Carus University Medical Center, TU Dresden, Dresden, Germany
Signatures of:	6a	Persons writing the SAP:<br><br>Matthias B. Moor May 11th 2022<br>Date and signature ....<br><br>Christoph Strumann<br>Date and signature May 11th 2022 ....

© 2022 Toepfner N et al. *JAMA Network Open.*

		Nicole Toepfner<br>Date and signature May 11th 2022 .... N. Töpfner
	6b	Senior statistician responsible:<br><br>Christoph Strumann<br>Date and signature May 11th 2022 ....
	6c	Chief investigator/clinical lead:<br><br>Cho-Ming Chao<br>Date and signature May 11th 2022 ....
Section 2: Introduction		
Background and rationale	7	Survey in parents regarding safety and side effects of the mRNA vaccine (BNT162b2) against COVID-19 in children below age of 5 years who were vaccinated (off-label use) in Germany. The aim of this study is to evaluate the tolerance and potential side effects of the mRNA-vaccine (BNT162b2) in these children.
Objectives	8	Primary objective is to analyze the rate of post-vaccination symptoms per vaccine dose, including as secondary objectives the time of onset, duration and severity or medical consequences of these symptoms. Secondary objectives are to evaluate these effects with regards to the number of given vaccines, the dosages of the vaccines, the potential recurrence of effects and age-dependent differences. A further subgroup analysis will comprise the evaluations of reported outcomes and effects in the children vaccinated according to the currently used vaccine strategy in children 5-11 years. As tertiary objectives, subgroup analysis of healthy children and children with underlying diseases will be performed and compared. In children with underlying diseases the reported vaccine-effects on the disease will be analyzed.<br>Reported outcomes will be compared to reported outcomes of non-SARS-CoV-2 vaccines in the same individuals.
Section 3: Study methods		
Study design	9	Retrospective cohort study.
Power considerations	10	For the primary analysis, 11 different categories of side effects (endpoints) are tested for differences between the 3 groups of dosages (3ug, 5ug and 10ug) by means of the Chi2 test. Setting the power for a single endpoint to 0.8 and the significance level to 0.05, and using the current number of completed questionnaires (n=4818), we obtain an effect size index of w = 0.0447 (Cohen, J., 2013).<br>This is regarded as a maximal bound of detectable differences. To take into account the correction for multiple testing by the Benferroni approach, the conjunctive power (i.e., the probability of finding a significant effect for all considered side effects) is improved by having more endpoints for low and moderate correlations between the endpoints (Senn and Bretz, 2007). If the overall power is fixed to be 0.8, this relates to a smaller w.
Framework	11	Superiority hypothesis testing of BNT162b2 vaccine safety.
Statistical interim analyses and stopping guidance	12a	No repetitive analyses. Technical interim analysis of missing data frequency on April 19th, 2022.
	12b	No planned adjustment of the significance level due to repetitive analyses

© 2022 Toepfner N et al. *JAMA Network Open.*

	12c	No plan for stopping the study early based on interim analyses.
Timing of final analysis	13	Database freezing May 9th, 2022 and full analysis.
Timing of outcome assessments	14	Survey time window April 14th to May 9th, 2022.
Section 4: Statistical principles		
Confidence intervals and P-values	15	Level of statistical significance: p=0.05 in Chi2, FET and ANOVA.
	16	Adjustment for multiplicity.
	17	Confidence interval to be reported: 95%
Adherence and protocol deviations	18a	Definition of protocol deviations for the trial: Change in outcomes or survey duration.
	18b	All protocol deviations as per 18a will be summarized.
Analysis populations	19	Only safety analysis in dosage and age subgroups, including an active comparator of non-SARS-CoV-2 vaccinations.
Section 5: Study Population		
Screening data	20	Numbers of eligible vaccination registrations will be reported.
Eligibility	21	Registrated child for SARS-CoV-2 vaccination before under age of 5 years in Germany at any time before April 14th, 2022.
Recruitment	22	Information to be included in the STROBE flow diagram: Number screened, number of replies, number of duplicates excluded, number of individuals included in analysis.
Withdrawal/follow-up	23a	Level of withdrawal or dropouts: NA
	23b	Timing of withdrawal/lost to follow-up data: NA
	23c	Reasons and details of how withdrawal/lost to follow-up: NA
Baseline patient characteristics	24a	List of baseline characteristics to be summarized for 1st, 2nd and 3rd BNT162b2 vaccination: Age and dosage of vaccines.<br>List of baseline characteristics summarized for 1st BNT162b2 vaccination: Age, gender, weight, height, number of vaccine doses, health status
	24b	Details of how baseline characteristics will be descriptively summarized: Median+IQR for non-normal distributed data. Mean+SD for normal distributed data. n/N (%) for proportions.
Potential confounding covariates	25	Age, weight, gender, dosage, chronic diseases, chronic medication, number of vaccine doses, comparison of BNT162b2 with non-SARS-CoV-2 vaccine: vaccine type (binary: «BNT162b2» vs «other») as covariate in logistic regression.
Section 6: Analysis		
Outcome definitions		List and describe each primary and secondary outcome including details of:<br>Primary outcomes: Self-reported symptom categories (yes/no) of local reactions, fever, skin, limb, pulmonary, cardiovascular, neurological, psychological, general, gastrointestinal, otorhinolaryngological

© 2022 Toepfner N et al. *JAMA Network Open*.

		symptoms, infections, number institutional missing days after vaccination.<br>Secondary outcomes: duration, severity and consequences of individual primary outcomes
	26a	Specification of outcomes and timings. If applicable include the order of importance of primary or key secondary end points (e.g., order in which they will be tested)
	26b	Specific measurement: n/N (%)
	26c	Any calculation or transformation used to derive the outcome: none.
Analysis methods	27a	What analysis method will be used and how the treatment effects will be presented: Frequency of symptom categories occurring in bar plot multi-panel and tabular data stratified by subgroups. Multivariate Logistic regression for each symptom categories and multivariate negative binomial regression for the number of symptoms.
	27b	Adjustment for covariates: age, gender, weight, BNT162b2 dosage, lot number, different vaccination platforms, chronic diseases, chronic medication
	27c	Methods used for assumptions to be checked for statistical methods: Descriptive stats of baseline variables and goodness of fit tests.
	27d	Details of alternative methods to be used if distributional assumptions do not hold: specification of non-linear and interaction effects as well as non- and semiparametric specifications to improve the fit of the logistic and negative binomial model.
	27e	Any planned sensitivity analyses for each outcome where applicable*
	27f	Any planned subgroup analyses for each outcome including how subgroups are defined:<br>Age categories 0-<12 months, 12-<24 months, >=24months to <5 years at first BNT162b2 vaccination.<br>Dosage categories of 3, 5, 10ug, unknown, other.
Missing data	28	Reporting and assumptions/statistical methods to handle missing data (e.g., multiple imputation)*<br>Reporting missing data as % in each variable.<br>Sensitivity analysis of full dataset vs verified-only dataset (incl. Dosage + BNT162b2 lot number)<br>Robustness check by Multiple Imputation. Complete variables serving as explanatory variables in the imputation models. Additional, aggregated geolocation dummy variables based on the postal code are used as regression variables in the imputation models if they obtain complete data.
Additional analyses	29	Details of any additional statistical analyses required: Matching children with different doses subject to age, gender, weight, lot number, different vaccination platforms, chronic diseases, chronic medication to increase the comparability.
Harms	30	Only applies when intervention effects are studied. Sufficient detail on summarizing safety data, e.g. information on severity, expectedness, and associations; details of how adverse events are scored; how adverse event data will be analysed and the follow-up time.
Statistical software	31	Details of statistical packages to be used to carry out analysis MATLAB R2020a and STATA 15

© 2022 Toepfner N et al. *JAMA Network Open.*

References	32a	References to be provided for nonstandard statistical methods:<br>Cohen, J. (2013). Statistical power analysis for the behavioral sciences. Routledge.<br>Senn, S., & Bretz, F. (2007). Power and sample size when multiple endpoints are considered. Pharmaceutical Statistics: The Journal of Applied Statistics in the Pharmaceutical Industry, 6(3), 161-170.
	32b	Reference to Data Management Plan: NA
	32c	Reference to the Study Master File and Statistical Master File: NA
	32d	Reference to other standard operation procedures to be adhered to: NA

Reference for template: https://doi.org/10.1186/s12874-019-0879-5

© 2022 Toepfner N et al. *JAMA Network Open*.

eAppendix 2. Supplementary Methods

Multiple imputation procedure

We assumed that data are missing at random enabling us to use other variables in the dataset to predict the missingness by chained equations (MICE). For the frequency analyses, we imputed the responses. For the logistic regression analysis, two comparative approaches have been applied. First, the multiple imputation is limited on the covariables with missing observations, i.e., height and weight. Second, additionally to the covariables the responses were imputed. For both approaches, we used 10 imputed datasets. The results of the imputed datasets were consolidated into one result by averaging the individual estimates for each of the imputed datasets. Standard errors are extracted from the covariance matrix, i.e., a combination of the within-imputation variance and the between-imputation variance [1]. In general, the estimated ORs of the BNT vaccine have been qualitatively very similar across the two different multiple imputation approaches.

eReference

1. White IR, Royston P, Wood AM. Multiple imputation using chained equations: Issues and guidance for practice. *Stat Med*. 2011;30(4):377-399. doi:https://doi.org/10.1002/sim.4067

eTable 1. Characteristics of Children Younger Than 5 Years Who Received the BNT162b2 Vaccination[a]

Characteristic	All	Vaccination		
		First	Second	Third
Sex				
Female	3824/7805 (49.0)	3824/7805 (49.0)	3482/7101 (49.0)	419/846 (49.5)
Male	3977/7805 (51.0)	3977/7805 (51.0)	3616/7101 (50.9)	427/846 (50.5)
Diverse	4/7805 (0.1)	4/7805 (0.1)	3/7101 (0.0)	0/846 (0.0)
Age, median (IQR), y	3 (2-4)	3 (2-4)	3 (2-4)	3 (2.5-4)
Height, median (IQR), cm	98 (88-105)	98 (88-105)	98 (88-105)	99 (92-105)
Weight, median (IQR), kg	14.5 (12-17)	14.5 (12-17)	14.5 (12-17)	15 (12.5-17)
BNT162b2, µg				
3	2995/14646 (20.4)	1772/7240 (24.5)	1171/6610 (17.7)	52/796 (6.5)
5	6075/14646 (41.5)	3120/7240 (43.1)	2740/6610 (41.5)	215/796 (27.0)
10	5576/14646 (38.1)	2348/7240 (32.4)	2699/6610 (40.8)	529/796 (66.5)
Comorbidities (yes)	684/7806 (8.8)	684/7806 (8.8)	613/7102 (8.6)	89/846 (10.5)
Long-term medication (yes)	381/7784 (4.9)	381/7784 (4.9)	337/7099 (4.7)	46/846 (5.4)

[a]Data are presented as number/total number (%) of study participants unless otherwise indicated.

© 2022 Toepfner N et al. *JAMA Network Open.*

eTable 2. Comorbidities, n (%)

Comorbidity	All<br>N=7784
None	7100 (91.2)
Pulmonary diseases	190 (2.4)
Malignant diseases	7 (0.1)
Rheumatological diseases	5 (0.1)
Gastroenterological diseases	17 (0.2)
Cardiovascular diseases	166 (2.1)
Immunological diseases	9 (0.1)
Trisomy 21	90 (1.2)
Other diseases	305 (3.9)

© 2022 Toepfner N et al. *JAMA Network Open.*

eTable 3. Post-vaccination symptoms after first BNT162b2 vaccine dose, n/N (%)

	<12 months				12 to <24 months				24 to <60 months			
Symptoms	3 µg	5 µg	10 µg	p-value*	3 µg	5 µg	10 µg	p-value*	3 µg	5 µg	10 µg	p-value*
Local	22/148 (14.9)	23/170 (13.5)	0/17 (0.0)	>0.999	110/515 (21.4)	154/651 (23.7)	29/97 (29.9)	>0.999	406/1094 (37.1)	806/2281 (35.3)	1023/2216 (46.2)	<0.001
General	17/148 (11.5)	21/170 (12.4)	1/17 (5.9)	>0.999	50/511 (9.8)	99/650 (15.2)	10/97 (10.3)	0.18	119/1090 (10.9)	277/2278 (12.2)	274/2208 (12.4)	>0.999
Fever	4/148 (2.7)	6/170 (3.5)	0/17 (0.0)	>0.999	19/511 (3.7)	33/650 (5.1)	5/97 (5.2)	>0.999	27/1090 (2.5)	86/2278 (3.8)	62/2208 (2.8)	0.74
Musculoskeletal	2/148 (1.4)	2/170 (1.2)	0/17 (0.0)	>0.999	3/511 (0.6)	11/649 (1.7)	1/97 (1.0)	>0.999	19/1086 (1.7)	65/2273 (2.9)	90/2201 (4.1)	0.010
Gastrointestinal	3/148 (2.0)	3/169 (1.8)	0/17 (0.0)	>0.999	7/512 (1.4)	14/647 (2.2)	2/96 (2.1)	>0.999	16/1088 (1.5)	42/2275 (1.8)	48/2200 (2.2)	>0.999
Otolaryngological	0/148 (0.0)	0/169 (0.0)	0/17 (0.0)	-	2/510 (0.4)	9/644 (1.4)	0/96 (0.0)	>0.999	13/1084 (1.2)	23/2271 (1.0)	25/2194 (1.1)	>0.999
Pulmonary	1/147 (0.7)	0/169 (0.0)	0/17 (0.0)	>0.999	8/511 (1.6)	8/643 (1.2)	1/96 (1)	>0.999	9/1085 (0.8)	20/2269 (0.9)	14/2195 (0.6)	>0.999
Cardiovascular	0/148 (0.0)	0/169 (0.0)	0/17 (0.0)	-	1/510 (0.2)	2/642 (0.3)	0/96 (0.0)	>0.999	2/1086 (0.2)	5/2268 (0.2)	7/2197 (0.3)	>0.999
Neurological	0/147 (0.0)	1/169 (0.6)	0/17 (0.0)	>0.999	1/510 (0.2)	3/642 (0.5)	0/96 (0.0)	>0.999	8/1081 (0.7)	19/2262 (0.8)	30/2191 (1.4)	>0.999
Psychological	4/148 (2.7)	2/169 (1.2)	0/17 (0.0)	>0.999	7/511 (1.4)	9/640 (1.4)	1/96 (1.0)	>0.999	9/1083 (0.8)	37/2264 (1.6)	24/2190 (1.1)	>0.999
Dermatological	2/148 (1.4)	1/169 (0.6)	0/17 (0.0)	>0.999	16/508 (3.1)	8/640 (1.3)	0/96 (0.0)	0.27	15/1084 (1.4)	31/2261 (1.4)	42/2183 (1.9)	>0.999

Notes: *Adjusted for multiple testing by Bonferroni correction.

© 2022 Toepfner N et al. *JAMA Network Open.*

eTable 4. Post-vaccination symptoms after second BNT162b2 vaccine dose, n/N (%)

	<12 months				12 to <24 months				24 to <60 months			
Symptoms	3 µg	5 µg	10 µg	p-value*	3 µg	5 µg	10 µg	p-value*	3 µg	5 µg	10 µg	p-value*
Local	8/108 (7.4)	26/162 (16.0)	0/20 (0.0)	0.26	68/378 (18.0)	145/628 (23.1)	38/141 (27)	0.55	222/676 (32.8)	640/1936 (33.1)	1024/2522 (40.6)	<0.001
General	11/108 (10.2)	17/162 (10.5)	1/20 (5)	>0.999	45/373 (12.1)	79/629 (12.6)	19/141 (13.5)	>0.999	68/675 (10.1)	197/1932 (10.2)	262/2516 (10.4)	>0.999
Fever	2/108 (1.9)	3/162 (1.9)	0/20 (0.0)	>0.999	23/373 (6.2)	46/629 (7.3)	8/141 (5.7)	>0.999	21/675 (3.1)	67/1932 (3.5)	89/2516 (3.5)	>0.999
Musculoskeletal	0/108 (0.0)	2/162 (1.2)	0/20 (0.0)	>0.999	4/373 (1.1)	9/628 (1.4)	1/141 (0.7)	>0.999	12/674 (1.8)	52/1926 (2.7)	96/2508 (3.8)	0.11
Gastrointestinal	2/108 (1.9)	0/161 (0.0)	0/20 (0.0)	>0.999	7/374 (1.9)	18/626 (2.9)	3/140 (2.1)	>0.999	11/674 (1.6)	36/1929 (1.9)	52/2505 (2.1)	>0.999
Otolaryngological	0/108 (0.0)	0/161 (0.0)	0/20 (0.0)	-	1/374 (0.3)	9/623 (1.4)	2/140 (1.4)	>0.999	5/672 (0.7)	24/1925 (1.2)	23/2501 (0.9)	>0.999
Pulmonary	1/107 (0.9)	0/161 (0.0)	0/20 (0.0)	>0.999	3/374 (0.8)	6/624 (1.0)	2/140 (1.4)	>0.999	1/671 (0.1)	10/1923 (0.5)	19/2502 (0.8)	>0.999
Cardiovascular	1/108 (0.9)	0/161 (0.0)	0/20 (0.0)	>0.999	0/373 (0.0)	2/623 (0.3)	0/139 (0.0)	>0.999	0/671 (0.0)	2/1925 (0.1)	9/2503 (0.4)	0.91
Neurological	0/107 (0.0)	1/161 (0.6)	0/20 (0.0)	>0.999	1/373 (0.3)	1/622 (0.2)	0/140 (0.0)	>0.999	4/668 (0.6)	14/1919 (0.7)	30/2498 (1.2)	>0.999
Psychological	2/108 (1.9)	2/161 (1.2)	0/20 (0.0)	>0.999	3/374 (0.8)	11/620 (1.8)	0/140 (0.0)	>0.999	6/669 (0.9)	28/1922 (1.5)	22/2496 (0.9)	>0.999
Dermatological	0/108 (0.0)	1/161 (0.6)	0/20 (0.0)	>0.999	9/372 (2.4)	14/620 (2.3)	5/140 (3.6)	>0.999	11/671 (1.6)	24/1916 (1.3)	62/2492 (2.5)	0.11

Notes: *Adjusted for multiple testing by Bonferroni correction.

© 2022 Toepfner N et al. *JAMA Network Open.*

eTable 5. Post-vaccination symptoms after third BNT162b2 vaccine dose, n/N (%)

	<12 months				12 to <24 months				24 to <60 months			
Symptoms	3 µg	5 µg	10 µg	p-value*	3 µg	5 µg	10 µg	p-value*	3 µg	5 µg	10 µg	p-value*
Local	0/3 (0.0)	3/11 (27.3)	1/4 (25.0)	>0.999	1/15 (6.7)	14/37 (37.8)	11/46 (23.9)	0.66	11/34 (32.4)	60/167 (35.9)	192/475 (40.4)	>0.999
General	1/3 (33.3)	2/11 (18.2)	1/4 (25.0)	>0.999	0/15 (0.0)	5/37 (13.5)	7/46 (15.2)	>0.999	2/34 (5.9)	17/167 (10.2)	60/475 (12.6)	>0.999
Fever	0/3 (0.0)	1/11 (9.1)	0/4 (0.0)	>0.999	0/15 (0.0)	2/37 (5.4)	3/46 (6.5)	>0.999	0/34 (0.0)	9/167 (5.4)	26/475 (5.5)	>0.999
Musculoskeletal	0/3 (0.0)	0/11 (0.0)	0/4 (0.0)	-	0/15 (0.0)	0/37 (0.0)	0/46 (0.0)	-	0/33 (0.0)	6/167 (3.6)	22/472 (4.7)	>0.999
Gastrointestinal	1/3 (33.3)	1/11 (9.1)	0/4 (0.0)	>0.999	0/15 (0.0)	1/37 (2.7)	1/46 (2.2)	>0.999	0/34 (0.0)	2/167 (1.2)	6/474 (1.3)	>0.999
Otolaryngological	0/3 (0.0)	0/11 (0.0)	0/4 (0.0)	-	0/15 (0.0)	0/37 (0.0)	1/46 (2.2)	>0.999	2/34 (5.9)	5/167 (3.0)	1/474 (0.2)	0.006
Pulmonary	0/3 (0.0)	1/11 (9.1)	0/4 (0.0)	>0.999	0/15 (0.0)	0/37 (0.0)	0/46 (0.0)	-	0/34 (0.0)	1/167 (0.6)	5/473 (1.1)	>0.999
Cardiovascular	0/3 (0.0)	0/11 (0.0)	0/4 (0.0)	-	0/15 (0.0)	0/37 (0.0)	0/46 (0.0)	-	0/34 (0.0)	0/167 (0.0)	1/472 (0.2)	>0.999
Neurological	0/3 (0.0)	0/11 (0.0)	0/4 (0.0)	-	0/15 (0.0)	0/37 (0.0)	2/46 (4.3)	>0.999	1/34 (2.9)	1/166 (0.6)	6/471 (1.3)	>0.999
Psychological	0/3 (0.0)	0/11 (0.0)	0/4 (0.0)	-	0/15 (0.0)	0/37 (0.0)	0/46 (0.0)	-	0/34 (0.0)	0/167 (0.0)	2/470 (0.4)	>0.999
Dermatological	0/3 (0.0)	1/11 (9.1)	0/4 (0.0)	>0.999	0/14 (0.0)	1/37 (2.7)	0/46 (0.0)	>0.999	1/34 (2.9)	3/167 (1.8)	17/471 (3.6)	>0.999

Notes: *Adjusted for multiple testing by Bonferroni correction.

© 2022 Toepfner N et al. *JAMA Network Open.*

eTable 6. Post-vaccination symptoms after any BNT162b2 vaccine dose, n/N (%)

	<12 months				12 to <24 months				24 to <60 months			
	3 µg	5 µg	10 µg	p-value	3 µg	5 µg	10 µg	p-value	3 µg	5 µg	10 µg	p-value
Number absent days (mean, N)	1/1	1/1	-/0	-	2/18	3.5/21	1.5/4	0.28	4.6/32	3/118	3/154	0.35
Susceptibility to infections	1/100 (1.0)	0/137 (0.0)	0/13 (0.0)	0.47	3/337 (0.9)	4/508 (0.8)	0/79 (0.0)	0.71	5/461 (1.1)	10/1532 (0.7)	20/1898 (1.1)	0.42
Threat rating scale (1-10), (mean, N)	0.1/99	0.1/135	0/13	0.84	0.1/332	0.2/499	0.1/79	0.25	0.1/454	0.1/1520	0.1/1885	0.92
Threat rating scale=0	94/99 (94.9)	131/135 (97.0)	13/13 (100)	0.54	307/332 (92.5)	457/499 (91.6)	75/79 (94.9)	0.57	417/454 (91.9)	1435/1520 (94.4)	1757/1885 (93.2)	0.11
Threat rating scale=1	3/99 (3.0)	1/135 (0.7)	0/13 (0.0)	0.35	21/332 (6.3)	22/499 (4.4)	3/79 (3.8)	0.40	29/454 (6.4)	54/1520 (3.6)	73/1885 (3.9)	0.02
Threat rating scale=2	1/99 (1.0)	1/135 (0.7)	0/13 (0.0)	0.92	1/332 (0.3)	13/499 (2.6)	0/79 (0.0)	0.02	6/454 (1.3)	15/1520 (1.0)	38/1885 (2.0)	0.05
Threat rating scale=3	1/99 (1.0)	1/135 (0.7)	0/13 (0.0)	0.92	2/332 (0.6)	2/499 (0.4)	1/79 (1.3)	0.62	1/454 (0.2)	7/1520 (0.5)	9/1885 (0.5)	0.75
Threat rating scale=4	0/99 (0.0)	0/135 (0.0)	0/13 (0.0)	-	0/332 (0.0)	2/499 (0.4)	0/79 (0.0)	0.44	1/454 (0.2)	2/1520 (0.1)	4/1885 (0.2)	0.84
Threat rating scale=5	0/99 (0.0)	0/135 (0.0)	0/13 (0.0)	-	1/332 (0.3)	2/499 (0.4)	0/79 (0.0)	0.84	0/454 (0.0)	3/1520 (0.2)	2/1885 (0.1)	0.55
Threat rating scale=6	0/99 (0.0)	0/135 (0.0)	0/13 (0.0)	-	0/332 (0.0)	1/499 (0.2)	0/79 (0.0)	0.66	0/454 (0.0)	1/1520 (0.1)	1/1885 (0.1)	0.86
Threat rating scale=7	0/99 (0.0)	1/135 (0.7)	0/13 (0.0)	0.66	0/332 (0.0)	0/499 (0.0)	0/79 (0.0)	-	0/454 (0.0)	0/1520 (0.0)	1/1885 (0.1)	0.59
Threat rating scale=8	0/99 (0.0)	0/135 (0.0)	0/13 (0.0)	-	0/332 (0.0)	0/499 (0.0)	0/79 (0.0)	-	0/454 (0.0)	0/1520 (0.0)	0/1885 (0.0)	-
Threat rating scale=9	0/99 (0.0)	0/135 (0.0)	0/13 (0.0)	-	0/332 (0.0)	0/499 (0.0)	0/79 (0.0)	-	0/454 (0.0)	1/1520 (0.1)	0/1885 (0.0)	0.46
Threat rating scale=10	0/99 (0.0)	0/135 (0.0)	0/13 (0.0)	-	0/332 (0.0)	0/499 (0.0)	0/79 (0.0)	-	0/454 (0.0)	2/1520 (0.1)	0/1885 (0.0)	0.21

Notes: *ANOVA. Threat rating scale ranging from 1 (non-threatening) to 10 (maximum imaginable threat to the life of the child).

© 2022 Toepfner N et al. *JAMA Network Open.*

eTable 7. Local symptoms

	Redness at the injection site	Swelling at the injection site	Pain at the injection site	Other local discomfort
After any vac.	934/3363 (27.8)	671/3363 (20)	2789/3363 (82.9)	52/3363 (1.5)
After 1st vac.	791/3363 (23.5)	530/3363 (15.8)	2276/3363 (67.7)	36/3363 (1.1)
After 2nd vac.	632/3363 (18.8)	448/3363 (13.3)	1915/3363 (56.9)	30/3363 (0.9)
Threat level (mean/N)	0.2±0.6/919	0.2±0.7/658	0.1±0.6/2749	0.2±0.6/52
Dosage				
3 µg	115/620 (18.5)	75/462 (16.2)	223/1838 (12.1)	5/30 (16.7)
5 µg	280/620 (45.2)	198/462 (42.9)	643/1838 (35)	8/30 (26.7)
10 µg	225/620 (36.3)	189/462 (40.9)	972/1838 (52.9)	17/30 (56.7)
mixed/unknown	314/934 (33.6)	209/671 (31.1)	951/2789 (34.1)	22/52 (42.3)
Age				
<12 months	32/934 (3.4)	21/671 (3.1)	27/2789 (1)	0/52 (0.0)
12 to <24 months	196/934 (21)	129/671 (19.2)	201/2789 (7.2)	5/52 (9.6)
24 to <60 months	706/934 (75.6)	521/671 (77.6)	2561/2789 (91.8)	47/52 (90.4)
Beginning & duration				
Beginning (days after vaccination, mean/N)	1.1±0.6/934	1±0.4/660	1±0.2/2764	1.1±0.7/52
Duration (days, mean/N)	2±1.8/915	2.4±3.9/651	1.6±1.1/2732	4.1±9.8/50
> 90 days	0/934 (0.0)	0/671 (0.0)	0/2789 (0.0)	0/52 (0.0)
Ongoing (days, mean/N)	0±0/0	0±0/0	0±0/0	93±0/1
unknown	6/934 (0.6)	4/671 (0.6)	12/2789 (0.4)	0/52 (0.0)
Disposition				
ambulatory	0/934 (0.0)	0/671 (0.0)	0/2789 (0.0)	1/52 (1.9)
inpatient	0/934 (0.0)	0/671 (0.0)	1/2789 (0.0)	0/52 (0.0)
mortality	0/934 (0.0)	0/671 (0.0)	0/2789 (0.0)	0/52 (0.0)
other	7/934 (0.7)	8/671 (1.2)	17/2789 (0.6)	1/52 (1.9)

Threat level from 0 (minimum) to 10 (maximum). Vac., vaccination. Ambulatory and inpatient refer to treatment requirement.

eTable 8. General symptoms

	Fever	Chills	Hot flashes	Fatigue	Flu-like symptoms	Feeling of weakness	malaise/general feeling of illness	MIS-C	Other general complaints
After any vac.	519/1505 (34.5)	56/1505 (3.7)	32/1505 (2.1)	1043/1505 (69.3)	131/1505 (8.7)	140/1505 (9.3)	342/1505 (22.7)	0/1505 (0.0)	100/1505 (6.6)
After 1st vac.	267/1505 (17.7)	28/1505 (1.9)	19/1505 (1.3)	766/1505 (50.9)	74/1505 (4.9)	100/1505 (6.6)	215/1505 (14.3)	0/1505 (0.0)	66/1505 (4.4)
After 2nd vac.	276/1505 (18.3)	28/1505 (1.9)	13/1505 (0.9)	606/1505 (40.3)	62/1505 (4.1)	67/1505 (4.5)	187/1505 (12.4)	0/1505 (0.0)	50/1505 (3.3)
Threat level (mean±SD/N)	0.6±1.1/513	0.9±1.5/55	0.2±0.5/31	0.3±0.9/1028	1±1.3/128	0.8±1.4/136	0.6±1.1/337	0±0/0	0.8±1.2/98
Dosage									
3 µg	49/330 (14.8)	4/37 (10.8)	4/23 (17.4)	104/689 (15.1)	15/83 (18.1)	12/93 (12.9)	41/233 (17.6)	0/0 (0.0)	7/61 (11.5)
5 µg	168/330 (50.9)	12/37 (32.4)	9/23 (39.1)	296/689 (43)	35/83 (42.2)	30/93 (32.3)	100/233 (42.9)	0/0 (0.0)	25/61 (41.0)
10 µg	113/330 (34.2)	21/37 (56.8)	10/23 (43.5)	289/689 (41.9)	33/83 (39.8)	51/93 (54.8)	92/233 (39.5)	0/0 (0.0)	29/61 (47.5)
mixed/unknown	189/519 (36.4)	19/56 (33.9)	9/32 (28.1)	354/1043 (33.9)	48/131 (36.6)	47/140 (33.6)	109/342 (31.9)	0/0 (0.0)	39/100 (39.0)
Age									
<12 months	15/519 (2.9)	0/56 (0.0)	2/32 (6.3)	37/1043 (3.5)	2/131 (1.5)	0/140 (0.0)	14/342 (4.1)	0/0 (0.0)	3/100 (3.0)
12 to <24 months	125/519 (24.1)	4/56 (7.1)	5/32 (15.6)	189/1043 (18.1)	24/131 (18.3)	17/140 (12.1)	68/342 (19.9)	0/0 (0.0)	13/100 (13.0)
24 to <60 months	379/519 (73.0)	52/56 (92.9)	25/32 (78.1)	817/1043 (78.3)	105/131 (80.2)	123/140 (87.9)	260/342 (76.0)	0/0 (0.0)	84/100 (84.0)
Beginning & duration									
beginning (days after vaccination, mean±SD/N)	1.1±0.4/519	1.1±0.3/56	1±0.2/31	1±0.2/1039	1.2±0.4/128	1.1±0.5/138	1±0.2/340	0±0/0	1.2±0.6/100
duration (days, mean±SD/N)	1.4±1.1/515	1.1±1/55	1±0.6/30	1.6±2.5/1026	2.9±2.4/128	2.3±2.9/138	1.9±1.7/337	0±0/0	2.8±4.5/99
> 90 days	0/519 (0.0)	0/56 (0.0)	0/32 (0.0)	0/1043 (0.0)	0/131 (0.0)	0/140 (0.0)	0/342 (0.0)	0/0 (0.0)	0/100 (0.0)
ongoing (days, mean±SD/N)	0±0/0	0±0/0	0±0/0	0±0/0	0±0/0	0±0/0	0±0/0	0±0/0	86±0/1

© 2022 Toepfner N et al. *JAMA Network Open.*

unknown	0/519 (0.0)	0/56 (0.0)	0/32 (0.0)	0/1043 (0.0)	0/131 (0.0)	0/140 (0.0)	0/342 (0.0)	0/0 (0.0)	0/100 (0.0)
Disposition									
ambulatory	13/519 (2.5)	1/56 (1.8)	0/32 (0.0)	4/1043 (0.4)	9/131 (6.9)	0/140 (0.0)	4/342 (1.2)	0/0 (0.0)	4/100 (4)
inpatient	0/519 (0.0)	0/56 (0.0)	0/32 (0.0)	1/1043 (0.1)	0/131 (0.0)	1/140 (0.7)	0/342 (0.0)	0/0 (0.0)	1/100 (1)
mortality	0/519 (0.0)	0/56 (0.0)	0/32 (0.0)	0/1043 (0.0)	0/131 (0.0)	0/140 (0.0)	0/342 (0.0)	0/0 (0.0)	0/100 (0.0)
other	1/519 (0.2)	0/56 (0.0)	0/32 (0.0)	3/1043 (0.3)	0/131 (0.0)	0/140 (0.0)	1/342 (0.3)	0/0 (0.0)	4/100 (4)

Threat level from 0 (minimum) to 10 (maximum). Vac., vaccination. Ambulatory and inpatient refer to treatment requirement. MIS-C, multisystem inflammatory syndrome in children.

© 2022 Toepfner N et al. *JAMA Network Open.*

eTable 9. Musculoskeletal symptoms

	muscle weakness	muscle pain	muscle twitching	neck/back pain	pain in the arms	pain in legs	joint pain	joint swelling	pain in limbs	other complaints
After any vac.	11/319 (3.4)	84/319 (26.3)	1/319 (0.3)	15/319 (4.7)	179/319 (56.1)	50/319 (15.7)	12/319 (3.8)	1/319 (0.3)	29/319 (9.1)	6/319 (1.9)
After 1st vac.	9/319 (2.8)	62/319 (19.4)	0/319 (0.0)	9/319 (2.8)	133/319 (41.7)	32/319 (10)	8/319 (2.5)	1/319 (0.3)	16/319 (5)	2/319 (0.6)
After 2nd vac.	6/319 (1.9)	53/319 (16.6)	1/319 (0.3)	10/319 (3.1)	110/319 (34.5)	26/319 (8.2)	6/319 (1.9)	0/319 (0.0)	13/319 (4.1)	4/319 (1.3)
Threat level (mean±SD/N)	2.1±3.2/9	0.4±0.9/82	7±0/1	0.9±1.7/15	0.2±0.8/174	0.5±1.4/49	1.4±1.7/12	1±0/1	1.1±1.5/28	1.8±1.5/6
Dosage										
3µg	2/8 (25.0)	3/48 (6.3)	0/1 (0.0)	0/9 (0.0)	7/106 (6.6)	5/34 (14.7)	0/7 (0.0)	0/1 (0.0)	2/19 (10.5)	1/3 (33.3)
5µg	3/8 (37.5)	25/48 (52.1)	1/1 (100)	5/9 (55.6)	29/106 (27.4)	16/34 (47.1)	2/7 (28.6)	0/1 (0.0)	4/19 (21.1)	1/3 (33.3)
10µg	3/8 (37.5)	20/48 (41.7)	0/1 (0.0)	4/9 (44.4)	70/106 (66.0)	13/34 (38.2)	5/7 (71.4)	1/1 (100)	13/19 (68.4)	1/3 (33.3)
mixed/unknown	3/11 (27.3)	36/84 (42.9)	0/1 (0.0)	6/15 (40.0)	73/179 (40.8)	16/50 (32.0)	5/12 (41.7)	0/1 (0.0)	10/29 (34.5)	3/6 (50.0)
Age										
<12 months	0/11 (0.0)	3/84 (3.6)	1/1 (100)	0/15 (0.0)	0/179 (0.0)	1/50 (2.0)	0/12 (0.0)	0/1 (0.0)	0/29 (0.0)	0/6 (0.0)
12 to <24 months	1/11 (9.1)	6/84 (7.1)	0/1 (0.0)	0/15 (0.0)	10/179 (5.6)	8/50 (16.0)	0/12 (0.0)	0/1 (0.0)	2/29 (6.9)	2/6 (33.3)
24 to <60 months	10/11 (90.9)	75/84 (89.3)	0/1 (0.0)	15/15 (100.0)	169/179 (94.4)	41/50 (82.0)	12/12 (100.0)	1/1 (100.0)	27/29 (93.1)	4/6 (66.7)
Beginning & duration										
beginning (days after vaccination, mean±SD/N)	1.3±0.6/11	1±0.2/83	3±0/1	1±0/15	1±0.1/178	1.5±1.1/50	1.8±0.8/12	3±0/1	1±0/29	1.5±0.8/6
duration (days, mean±SD/N)	4.3±6.5/10	2.1±1.4/80	10±0/1	2.6±2.3/15	1.8±1.5/176	2.3±2.1/44	4±3.8/11	4±0/1	1.9±1.5/28	1.9±1.8/5
> 90 days	0/11 (0.0)	0/84 (0.0)	0/1 (0.0)	0/15 (0.0)	0/179 (0.0)	0/50 (0.0)	0/12 (0.0)	0/1 (0.0)	0/29 (0.0)	0/6 (0.0)

© 2022 Toepfner N et al. *JAMA Network Open.*

ongoing (days, mean±SD/N)	0±0/0	86±0/1	0±0/0	0±0/0	0±0/0	61±43.7/4	41±0/1	0±0/0	0±0/0	86±0/1
unknown	0/11 (0.0)	0/84 (0.0)	0/1 (0.0)	0/15 (0.0)	0/179 (0.0)	0/50 (0.0)	0/12 (0.0)	0/1 (0.0)	1/29 (3.4)	0/6 (0.0)
Disposition										
ambulatory	1/11 (9.1)	1/84 (1.2)	0/1 (0.0)	1/15 (6.7)	0/179 (0.0)	2/50 (4)	3/12 (25)	1/1 (100)	1/29 (3.4)	0/6 (0.0)
inpatient	1/11 (9.1)	0/84 (0.0)	1/1 (100)	0/15 (0.0)	0/179 (0.0)	0/50 (0.0)	0/12 (0.0)	0/1 (0.0)	0/29 (0.0)	0/6 (0.0)
mortality	0/11 (0.0)	0/84 (0.0)	0/1 (0.0)	0/15 (0.0)	0/179 (0.0)	0/50 (0.0)	0/12 (0.0)	0/1 (0.0)	0/29 (0.0)	0/6 (0.0)
other	0/11 (0.0)	0/84 (0.0)	0/1 (0.0)	0/15 (0.0)	1/179 (0.6)	1/50 (2)	1/12 (8.3)	0/1 (0.0)	0/29 (0.0)	0/6 (0.0)

Threat level from 0 (minimum) to 10 (maximum). Vac., vaccination. Ambulatory and inpatient refer to treatment requirement.

© 2022 Toepfner N et al. *JAMA Network Open.*

eTable 10. Gastrointestinal symptoms

	abdominal pain	nausea/vomiting	constipation	diarrhea	other stool changes	unwanted weight loss	unwanted weight gain	other gastrointestinal complaints
After any vac.	61/259 (23.6)	108/259 (41.7)	14/259 (5.4)	82/259 (31.7)	45/259 (17.4)	2/259 (0.8)	0/259 (0.0)	7/259 (2.7)
After 1st vac.	31/259 (12.0)	60/259 (23.2)	6/259 (2.3)	50/259 (19.3)	25/259 (9.7)	0/259 (0.0)	0/259 (0.0)	0/259 (0.0)
After 2nd vac.	29/259 (11.2)	50/259 (19.3)	10/259 (3.9)	47/259 (18.1)	32/259 (12.4)	2/259 (0.8)	0/259 (0.0)	5/259 (1.9)
Threat level (mean±SD/N)	1.4±1.5/60	0.9±1.3/106	0.8±1.3/14	0.9±1.4/82	0.5±0.9/44	4±0/2	0±0/0	2±1.6/7
Dosage								
3 µg	7/39 (17.9)	8/73 (11.0)	3/8 (37.5)	4/50 (8.0)	7/31 (22.6)	0/2 (0.0)	0/0 (0.0)	1/4 (25.0)
5 µg	10/39 (25.6)	35/73 (47.9)	3/8 (37.5)	24/50 (48.0)	12/31 (38.7)	2/2 (100)	0/0 (0.0)	2/4 (50.0)
10 µg	22/39 (56.4)	30/73 (41.1)	2/8 (25.0)	22/50 (44.0)	12/31 (38.7)	0/2 (0.0)	0/0 (0.0)	1/4 (25.0)
mixed/unknown	22/61 (36.1)	35/108 (32.4)	6/14 (42.9)	32/82 (39.0)	14/45 (31.1)	0/2 (0.0)	0/0 (0.0)	3/7 (42.9)
Age								
<12 months	1/61 (1.6)	1/108 (0.9)	0/14 (0.0)	4/82 (4.9)	4/45 (8.9)	0/2 (0.0)	0/0 (0.0)	1/7 (14.3)
12 to <24 months	3/61 (4.9)	18/108 (16.7)	3/14 (21.4)	17/82 (20.7)	9/45 (20.0)	2/2 (100.0)	0/0 (0.0)	0/7 (0.0)
24 to <60 months	57/61 (93.4)	89/108 (82.4)	11/14 (78.6)	61/82 (74.4)	32/45 (71.1)	0/2 (0.0)	0/0 (0.0)	6/7 (85.7)
Beginning & duration								
beginning (days after vaccination, mean±SD/N)	1.6±1/60	1.2±0.6/108	2.1±1.8/14	1.5±0.8/82	1.3±0.9/45	1±0/2	0±0/0	2.2±1.6/6
duration (days, mean±SD/N)	4.6±9.4/53	1.3±1.6/108	11.2±10.7/13	4.3±7.8/81	4.3±7/45	4±1.4/2	0±0/0	2±1/3
> 90 days	0/61 (0.0)	0/108 (0.0)	0/14 (0.0)	0/82 (0.0)	0/45 (0.0)	0/2 (0.0)	0/0 (0.0)	0/7 (0.0)
ongoing (days, mean±SD/N)	45.7±38.2/3	0±0/0	55±0/1	0±0/0	0±0/0	0±0/0	0±0/0	79±53.7/2
unknown	2/61 (3.3)	0/108 (0.0)	0/14 (0.0)	0/82 (0.0)	0/45 (0.0)	0/2 (0.0)	0/0 (0.0)	0/7 (0.0)
Disposition								

© 2022 Toepfner N et al. *JAMA Network Open.*

ambulatory	4/61 (6.6)	1/108 (0.9)	1/14 (7.1)	7/82 (8.5)	2/45 (4.4)	0/2 (0.0)	0/0 (0.0)	1/7 (14.3)
inpatient	1/61 (1.6)	0/108 (0.0)	0/14 (0.0)	0/82 (0.0)	0/45 (0.0)	0/2 (0.0)	0/0 (0.0)	0/7 (0.0)
mortality	0/61 (0.0)	0/108 (0.0)	0/14 (0.0)	0/82 (0.0)	0/45 (0.0)	0/2 (0.0)	0/0 (0.0)	0/7 (0.0)
other	0/61 (0.0)	2/108 (1.9)	0/14 (0.0)	1/82 (1.2)	0/45 (0.0)	0/2 (0.0)	0/0 (0.0)	1/7 (14.3)

Threat level from 0 (minimum) to 10 (maximum). Vac., vaccination. Ambulatory and inpatient refer to treatment requirement.

© 2022 Toepfner N et al. *JAMA Network Open.*

eTable 11. Otolaryngological symptoms

	Nosebleed	redness of the oral mucosa	swelling of the tongue	swelling of the lips	discomfort in the mouth	toothache	bleeding of the gums	tightness in the throat	sore throat	earache	hoarseness	facial swelling	swollen lymph nodes	painful lymph nodes	olfactory disorder	taste disorder	other complaints
After any vac.	17/132 (12.9)	4/132 (3)	0/132 (0.0)	0/132 (0.0)	1/132 (0.8)	6/132 (4.5)	0/132 (0.0)	2/132 (1.5)	20/132 (15.2)	25/132 (18.9)	13/132 (9.8)	2/132 (1.5)	44/132 (33.3)	5/132 (3.8)	1/132 (0.8)	2/132 (1.5)	18/132 (13.6)
After 1st vac.	10/132 (7.6)	3/132 (2.3)	0/132 (0.0)	0/132 (0.0)	0/132 (0.0)	2/132 (1.5)	0/132 (0.0)	2/132 (1.5)	12/132 (9.1)	12/132 (9.1)	5/132 (3.8)	0/132 (0.0)	29/132 (22.0)	3/132 (2.3)	1/132 (0.8)	1/132 (0.8)	11/132 (8.3)
After 2nd vac.	9/132 (6.8)	2/132 (1.5)	0/132 (0.0)	0/132 (0.0)	1/132 (0.8)	5/132 (3.8)	0/132 (0.0)	0/132 (0.0)	9/132 (6.8)	13/132 (9.8)	6/132 (4.5)	2/132 (1.5)	28/132 (21.2)	2/132 (1.5)	0/132 (0.0)	1/132 (0.8)	7/132 (5.3)
Threat level (mean±SD/N)	0.8±1.3/17	0.8±1/4	0±0/0	0±0/0	2±0/1	1±2/6	0±0/0	3±1.4/2	1.7±1.8/20	1.2±1.4/25	1.1±1.7/13	1.5±2.1/2	0.6±1.2/42	0.2±0.4/5	1±0/1	1.5±0.7/2	0.6±0.8/18
Dosage																	
3 µg	0/8 (0.0)	0/3 (0.0)	0/0 (0.0)	0/0 (0.0)	0/1 (0.0)	0/4 (0.0)	0/0 (0.0)	0/2 (0.0)	3/15 (20.0)	3/14 (21.4)	0/9 (0.0)	0/1 (0.0)	2/27 (7.4)	0/4 (0.0)	0/0 (0.0)	0/0 (0.0)	2/12 (16.7)
5 µg	4/8 (50.0)	2/3 (66.7)	0/0 (0.0)	0/0 (0.0)	0/1 (0.0)	3/4 (75.0)	0/0 (0.0)	1/2 (50.0)	6/15 (40.0)	5/14 (35.7)	6/9 (66.7)	1/1 (100.0)	13/27 (48.1)	0/4 (0.0)	0/0 (0.0)	0/0 (0.0)	6/12 (50.0)
10 µg	4/8 (50.0)	1/3 (33.3)	0/0 (0.0)	0/0 (0.0)	1/1 (100.0)	1/4 (25.0)	0/0 (0.0)	1/2 (50.0)	6/15 (40.0)	6/14 (42.9)	3/9 (33.3)	0/1 (0.0)	12/27 (44.4)	4/4 (100.0)	0/0 (0.0)	0/0 (0.0)	4/12 (33.3)
mixed/unknown	9/17 (52.9)	1/4 (25.0)	0/0 (0.0)	0/0 (0.0)	0/1 (0.0)	2/6 (33.3)	0/0 (0.0)	0/2 (0.0)	5/20 (25.0)	11/25 (44.0)	4/13 (30.8)	1/2 (50.0)	17/44 (38.6)	1/5 (20.0)	1/1 (100.0)	2/2 (100.0)	6/18 (33.3)
Age																	
<12 months	0/17 (0.0)	0/4 (0.0)	0/0 (0.0)	0/0 (0.0)	0/1 (0.0)	0/6 (0.0)	0/0 (0.0)	0/2 (0.0)	0/20 (0.0)	0/25 (0.0)	0/13 (0.0)	0/2 (0.0)	0/44 (0.0)	0/5 (0.0)	0/1 (0.0)	0/2 (0.0)	1/18 (5.6)
12 to <24 months	1/17 (5.9)	0/4 (0.0)	0/0 (0.0)	0/0 (0.0)	0/1 (0.0)	3/6 (50.0)	0/0 (0.0)	0/2 (0.0)	4/20 (20.0)	4/25 (16.0)	3/13 (23.1)	0/2 (0.0)	7/44 (15.9)	0/5 (0.0)	0/1 (0.0)	0/2 (0.0)	2/18 (11.1)

© 2022 Toepfner N et al. *JAMA Network Open.*

24 to <60 months	16/17 (94.1)	4/4 (100)	0/0 (0.0)	0/0 (0.0)	1/1 (100)	3/6 (50.0)	0/0 (0.0)	2/2 (100.0)	16/20 (80.0)	21/25 (84.0)	10/13 (76.9)	2/2 (100.0)	37/44 (84.1)	5/5 (100.0)	1/1 (100.0)	2/2 (100.0)	15/18 (83.3)
beginning & duration																	
beginning (days after vaccination, mean±SD/N)	2.2±1.6/17	1.5±0.6/4	0±0/0	0±0/0	1±0/1	2.2±1.6/6	0±0/0	1±0/2	1.5±0.7/20	1.6±0.9/25	1.4±0.7/12	1±0/2	1.6±1.2/44	1.2±0.4/5	1±0/1	2±0/2	2.6±2/18
duration (days, mean±SD/N)	1.9±2.2/17	4.5±1.7/4	0±0/0	0±0/0	1±0/1	5.2±1.8/6	0±0/0	3±0/2	3.4±2/19	2.3±1.6/23	2.3±1.2/11	1.3±1.1/2	5.3±9/43	2±0.8/4	16±0/1	9±7.1/2	12±23.2/13
> 90 days	0/17 (0.0)	0/4 (0.0)	0/0 (0.0)	0/0 (0.0)	0/1 (0.0)	0/6 (0.0)	0/0 (0.0)	0/2 (0.0)	0/20 (0.0)	0/25 (0.0)	0/13 (0.0)	0/2 (0.0)	0/44 (0.0)	0/5 (0.0)	0/1 (0.0)	0/2 (0.0)	0/18 (0.0)
ongoing (days, mean±SD/N)	0±0/0	0±0/0	0±0/0	0±0/0	0±0/0	0±0/0	0±0/0	0±0/0	0±0/0	41±0/1	0±0/0	0±0/0	0±0/0	0±0/0	0±0/0	0±0/0	57±23.7/4
unknown	0/17 (0.0)	0/4 (0.0)	0/0 (0.0)	0/0 (0.0)	0/1 (0.0)	0/6 (0.0)	0/0 (0.0)	0/2 (0.0)	0/20 (0.0)	0/25 (0.0)	0/13 (0.0)	0/2 (0.0)	0/44 (0.0)	0/5 (0.0)	0/1 (0.0)	0/2 (0.0)	1/18 (5.6)
Disposition																	
ambulatory	0/17 (0.0)	0/4 (0.0)	0/0 (0.0)	0/0 (0.0)	0/1 (0.0)	0/6 (0.0)	0/0 (0.0)	0/2 (0.0)	5/20 (25.0)	10/25 (40.0)	1/13 (7.7)	0/2 (0.0)	0/44 (0.0)	0/5 (0.0)	0/1 (0.0)	0/2 (0.0)	6/18 (33.3)
inpatient	0/17 (0.0)	0/4 (0.0)	0/0 (0.0)	0/0 (0.0)	0/1 (0.0)	0/6 (0.0)	0/0 (0.0)	0/2 (0.0)	0/20 (0.0)	0/25 (0.0)	0/13 (0.0)	0/2 (0.0)	1/44 (2.3)	0/5 (0.0)	0/1 (0.0)	0/2 (0.0)	0/18 (0.0)
mortality	0/17 (0.0)	0/4 (0.0)	0/0 (0.0)	0/0 (0.0)	0/1 (0.0)	0/6 (0.0)	0/0 (0.0)	0/2 (0.0)	0/20 (0.0)	0/25 (0.0)	0/13 (0.0)	0/2 (0.0)	0/44 (0.0)	0/5 (0.0)	0/1 (0.0)	0/2 (0.0)	0/18 (0.0)
other	0/17 (0.0)	0/4 (0.0)	0/0 (0.0)	0/0 (0.0)	0/1 (0.0)	0/6 (0.0)	0/0 (0.0)	0/2 (0.0)	1/20 (5.0)	1/25 (4.0)	0/13 (0.0)	1/2 (50)	0/44 (0.0)	0/5 (0.0)	0/1 (0.0)	0/2 (0.0)	2/18 (11.1)

Threat level from 0 (minimum) to 10 (maximum). Vac., vaccination. Ambulatory and inpatient refer to treatment requirement.

© 2022 Toepfner N et al. *JAMA Network Open.*

eTable 12. Pulmonary symptoms

	cough	irregular breathing	rapid breathing	shortness of breath on exertion	dyspnea at rest	other breathing problems
After any vac.	88/104 (84.6)	2/104 (1.9)	7/104 (6.7)	6/104 (5.8)	7/104 (6.7)	13/104 (12.5)
After 1st vac.	55/104 (52.9)	2/104 (1.9)	4/104 (3.8)	5/104 (4.8)	5/104 (4.8)	8/104 (7.7)
After 2nd vac.	36/104 (34.6)	0/104 (0.0)	3/104 (2.9)	1/104 (1)	1/104 (1)	5/104 (4.8)
Threat level (mean/N)	1.2±1.6/86	0.5±0.7/2	2.1±1.6/7	2.2±2.4/6	2.1±2.5/7	2.8±2.1/13
Dosage						
3 µg	8/57 (14.0)	0/2 (0.0)	1/3 (33.3)	0/3 (0.0)	0/5 (0.0)	1/9 (11.1)
5 µg	25/57 (43.9)	1/2 (50)	1/3 (33.3)	2/3 (66.7)	1/5 (20.0)	5/9 (55.6)
10 µg	24/57 (42.1)	1/2 (50)	1/3 (33.3)	1/3 (33.3)	4/5 (80.0)	3/9 (33.3)
mixed/unknown	31/88 (35.2)	0/2 (0.0)	4/7 (57.1)	3/6 (50)	2/7 (28.6)	4/13 (30.8)
Age						
<12 months	2/88 (2.3)	0/2 (0.0)	0/7 (0.0)	0/6 (0.0)	1/7 (14.3)	0/13 (0.0)
12 to <24 months	19/88 (21.6)	0/2 (0.0)	1/7 (14.3)	2/6 (33.3)	0/7 (0.0)	3/13 (23.1)
24 to <60 months	67/88 (76.1)	2/2 (100.0)	6/7 (85.7)	4/6 (66.7)	6/7 (85.7)	10/13 (76.9)
Beginning &duration						
beginning (days after vaccination, mean/N)	1.5±0.9/86	2±0/2	1.1±0.4/7	2.3±2/6	1.7±0.8/7	1.2±0.4/13
duration (days, mean/N)	5.7±4.2/82	8±8.5/2	7.9±10.7/7	8.8±7.3/5	3.8±3.6/6	4±3.1/12
> 90 days	0/88 (0.0)	0/2 (0.0)	0/7 (0.0)	0/6 (0.0)	0/7 (0.0)	0/13 (0.0)
ongoing (days, mean/N)	18±21.2/2	0±0/0	0±0/0	0±0/0	0±0/0	3±0/1
unknown	0/88 (0.0)	0/2 (0.0)	0/7 (0.0)	0/6 (0.0)	0/7 (0.0)	0/13 (0.0)
Disposition						
ambulatory	9/88 (10.2)	0/2 (0.0)	2/7 (28.6)	1/6 (16.7)	0/7 (0.0)	4/13 (30.8)
inpatient	2/88 (2.3)	1/2 (50.0)	2/7 (28.6)	2/6 (33.3)	3/7 (42.9)	3/13 (23.1)
mortality	0/88 (0.0)	0/2 (0.0)	0/7 (0.0)	0/6 (0.0)	0/7 (0.0)	0/13 (0.0)
other	2/88 (2.3)	0/2 (0.0)	0/7 (0.0)	0/6 (0.0)	1/7 (14.3)	2/13 (15.4)

Threat level from 0 (minimum) to 10 (maximum). Vac., vaccination. Ambulatory and inpatient refer to treatment requirement.

© 2022 Toepfner N et al. *JAMA Network Open.*

eTable 13. Cardiovascular symptoms

	syncope	tachycardia	heart pain	chest tightness	cold hands and feet	other
After any vac.	1/32 (3.1)	11/32 (34.4)	5/32 (15.6)	1/32 (3.1)	12/32 (37.5)	2/32 (6.3)
After 1st vac.	0/32 (0.0)	3/32 (9.4)	3/32 (9.4)	1/32 (3.1)	10/32 (31.3)	0/32 (0.0)
After 2nd vac.	1/32 (3.1)	8/32 (25.0)	3/32 (9.4)	0/32 (0.0)	2/32 (6.3)	2/32 (6.3)
Threat level (mean/N)	6±0/1	2.3±2.7/11	0.6±1.3/5	3±0/1	1.3±2.2/12	3±1.4/2
Dosage						
3 µg	0/1 (0.0)	1/9 (11.1)	1/5 (20.0)	0/0 (0.0)	1/9 (11.1)	0/2 (0.0)
5 µg	0/1 (0.0)	3/9 (33.3)	1/5 (20.0)	0/0 (0.0)	5/9 (55.6)	0/2 (0.0)
10 µg	1/1 (100.0)	5/9 (55.6)	3/5 (60.0)	0/0 (0.0)	3/9 (33.3)	2/2 (100.0)
mixed/unknown	0/1 (0.0)	2/11 (18.2)	0/5 (0.0)	1/1 (100.0)	3/12 (25.0)	0/2 (0.0)
Age						
<12 months	0/1 (0.0)	1/11 (9.1)	0/5 (0.0)	0/1 (0.0)	0/12 (0.0)	0/2 (0.0)
12 to <24 months	0/1 (0.0)	0/11 (0.0)	1/5 (20.0)	0/1 (0.0)	3/12 (25.0)	0/2 (0.0)
24 to <60 months	1/1 (100.0)	10/11 (90.9)	4/5 (80.0)	1/1 (100.0)	9/12 (75.0)	2/2 (100)
Beginning & duration						
beginning (days after vaccination, mean/N)	3±0/1	1.5±0.9/11	1.8±0.4/5	1±0/1	1.3±0.6/12	1.5±0.7/2
duration (days, mean/N)	0.5±0/1	2.7±4/10	3.5±2.5/5	0.5±0/1	3.1±2.4/10	0.8±0.4/2
> 90 days	0/1 (0.0)	0/11 (0.0)	0/5 (0.0)	0/1 (0.0)	0/12 (0.0)	0/2 (0.0)
ongoing (days, mean/N)	0±0/0	32±0/1	0±0/0	0±0/0	0±0/0	0±0/0
unknown	0/1 (0.0)	0/11 (0.0)	0/5 (0.0)	0/1 (0.0)	0/12 (0.0)	0/2 (0.0)
Disposition						
ambulatory	0/1 (0.0)	3/11 (27.3)	0/5 (0.0)	0/1 (0.0)	0/12 (0.0)	0/2 (0.0)
inpatient	1/1 (100.0)	1/11 (9.1)	0/5 (0.0)	0/1 (0.0)	0/12 (0.0)	0/2 (0.0)
mortality	0/1 (0.0)	0/11 (0.0)	0/5 (0.0)	0/1 (0.0)	0/12 (0.0)	0/2 (0.0)
other	0/1 (0.0)	0/11 (0.0)	0/5 (0.0)	0/1 (0.0)	0/12 (0.0)	0/2 (0.0)

Threat level from 0 (minimum) to 10 (maximum). Vac., vaccination. Ambulatory and inpatient refer to treatment requirement

© 2022 Toepfner N et al. *JAMA Network Open.*

eTable 14. Neurological symptoms

	headache	dizziness	sensory disturbances	locomotor disorders	loss of consciousness	shooting pain	seizure	paralysis of facial muscles	other complaints
After any vac.	101/121 (83.5)	12/121 (9.9)	1/121 (0.8)	5/121 (4.1)	1/121 (0.8)	1/121 (0.8)	1/121 (0.8)	0/121 (0.0)	3/121 (2.5)
After 1st vac.	54/121 (44.6)	6/121 (5)	1/121 (0.8)	3/121 (2.5)	1/121 (0.8)	0/121 (0.0)	0/121 (0.0)	0/121 (0.0)	1/121 (0.8)
After 2nd vac.	44/121 (36.4)	4/121 (3.3)	0/121 (0.0)	3/121 (2.5)	0/121 (0.0)	1/121 (0.8)	1/121 (0.8)	0/121 (0.0)	3/121 (2.5)
Threat level (mean±SD/N)	0.5±0.9/101	2.2±3/12	1±0/1	2.2±2.3/5	9±0/1	1±0/1	1±0/1	0±0/0	4±3/3
Dosage									
3 µg	9/64 (14.1)	1/10 (10.0)	0/1 (0.0)	0/4 (0.0)	0/1 (0.0)	0/1 (0.0)	0/1 (0.0)	0/0 (0.0)	0/1 (0.0)
5 µg	16/64 (25.0)	5/10 (50.0)	0/1 (0.0)	3/4 (75.0)	1/1 (100.0)	0/1 (0.0)	1/1 (100.0)	0/0 (0.0)	1/1 (100.0)
10 µg	39/64 (60.9)	4/10 (40.0)	1/1 (100.0)	1/4 (25.0)	0/1 (0.0)	1/1 (100.0)	0/1 (0.0)	0/0 (0.0)	0/1 (0.0)
mixed/unknown	37/101 (36.6)	2/12 (16.7)	0/1 (0.0)	1/5 (20.0)	0/1 (0.0)	0/1 (0.0)	0/1 (0.0)	0/0 (0.0)	2/3 (66.7)
Age									
<12 months	1/101 (1.0)	0/12 (0.0)	0/1 (0.0)	0/5 (0.0)	0/1 (0.0)	0/1 (0.0)	0/1 (0.0)	0/0 (0.0)	1/3 (33.3)
12 to <24 months	6/101 (5.9)	1/12 (8.3)	0/1 (0.0)	2/5 (40.0)	0/1 (0.0)	0/1 (0.0)	0/1 (0.0)	0/0 (0.0)	0/3 (0.0)
24 to <60 months	94/101 (93.1)	11/12 (91.7)	1/1 (100)	3/5 (60.0)	1/1 (100.0)	1/1 (100.0)	1/1 (100.0)	0/0 (0.0)	2/3 (66.7)
Beginning & duration									
beginning (days after vaccination, mean±SD/N)	1.1±0.5/95	2.2±1.9/12	3±0/1	2.4±2.2/5	3±0/1	5±0/1	1±0/1	0±0/0	2.3±1.2/3
duration (days, mean±SD/N)	1.6±2.1/94	2.5±3.4/11	0±0/0	2.3±2.8/5	1±0/1	6±0/1	0.5±0/1	0±0/0	21±0/1
> 90 days	0/101 (0.0)	0/12 (0.0)	1/1 (100)	0/5 (0.0)	0/1 (0.0)	0/1 (0.0)	0/1 (0.0)	0/0 (0.0)	0/3 (0.0)
ongoing (days, mean±SD/N)	66±0/1	0±0/0	0±0/0	0±0/0	0±0/0	0±0/0	0±0/0	0±0/0	86±0/1
unknown	0/101 (0.0)	0/12 (0.0)	0/1 (0.0)	0/5 (0.0)	0/1 (0.0)	0/1 (0.0)	0/1 (0.0)	0/0 (0.0)	0/3 (0.0)

© 2022 Toepfner N et al. *JAMA Network Open.*

Disposition									
ambulatory	0/101 (0.0)	1/12 (8.3)	0/1 (0.0)	0/5 (0.0)	0/1 (0.0)	0/1 (0.0)	0/1 (0.0)	0/0 (0.0)	0/3 (0.0)
inpatient	1/101 (1.0)	2/12 (16.7)	0/1 (0.0)	1/5 (20.0)	1/1 (100.0)	0/1 (0.0)	0/1 (0.0)	0/0 (0.0)	1/3 (33.3)
mortality	0/101 (0.0)	0/12 (0.0)	0/1 (0.0)	0/5 (0.0)	0/1 (0.0)	0/1 (0.0)	0/1 (0.0)	0/0 (0.0)	0/3 (0.0)
other	1/101 (1.0)	0/12 (0.0)	0/1 (0.0)	0/5 (0.0)	0/1 (0.0)	0/1 (0.0)	1/1 (100.0)	0/0 (0.0)	0/3 (0.0)

Threat level from 0 (minimum) to 10 (maximum). Vac., vaccination. Ambulatory and inpatient refer to treatment requirement.

.

© 2022 Toepfner N et al. *JAMA Network Open.*

eTable 15. Psychological symptoms

	concentration disorders	memory disorder	sleep disorders	aggressive behavior	anxiousness	hyperactivity	timid behavior	sadness/ depression	mood swings	altered behavior or other complaints
After any vac.	4/136 (2.9)	1/136 (0.7)	40/136 (29.4)	10/136 (7.4)	5/136 (3.7)	41/136 (30.1)	12/136 (8.8)	7/136 (5.1)	30/136 (22.1)	16/136 (11.8)
After 1st vac.	4/136 (2.9)	0/136 (0.0)	30/136 (22.1)	7/136 (5.1)	3/136 (2.2)	36/136 (26.5)	7/136 (5.1)	3/136 (2.2)	19/136 (14.0)	11/136 (8.1)
After 2nd vac.	2/136 (1.5)	0/136 (0.0)	26/136 (19.1)	7/136 (5.1)	5/136 (3.7)	23/136 (16.9)	9/136 (6.6)	3/136 (2.2)	18/136 (13.2)	8/136 (5.9)
Threat level (mean±SD/N)	0±0/4	1±0/1	0.3±0.6/40	1±1.2/10	1.4±3.1/5	0.2±0.7/41	0.8±1.3/12	2.1±3/7	0.8±1.5/30	0.8±1.1/16
Dosage										
3 µg	0/3 (0.0)	0/1 (0.0)	3/27 (11.1)	2/7 (28.6)	0/3 (0.0)	5/30 (16.7)	1/7 (14.3)	0/5 (0.0)	3/19 (15.8)	0/9 (0.0)
5 µg	2/3 (66.7)	0/1 (0.0)	16/27 (59.3)	3/7 (42.9)	2/3 (66.7)	16/30 (53.3)	4/7 (57.1)	2/5 (40.0)	11/19 (57.9)	8/9 (88.9)
10 µg	1/3 (33.3)	1/1 (100.0)	8/27 (29.6)	2/7 (28.6)	1/3 (33.3)	9/30 (30.0)	2/7 (28.6)	3/5 (60.0)	5/19 (26.3)	1/9 (11.1)
mixed/unknown	1/4 (25.0)	0/1 (0.0)	13/40 (32.5)	3/10 (30.0)	2/5 (40.0)	11/41 (26.8)	5/12 (41.7)	2/7 (28.6)	11/30 (36.7)	7/16 (43.8)
Age										
<12 months	0/4 (0.0)	0/1 (0.0)	4/40 (10.0)	0/10 (0.0)	1/5 (20.0)	2/41 (4.9)	0/12 (0.0)	1/7 (14.3)	2/30 (6.7)	1/16 (6.3)
12 to <24 months	0/4 (0.0)	0/1 (0.0)	14/40 (35.0)	1/10 (10.0)	0/5 (0.0)	5/41 (12.2)	1/12 (8.3)	0/7 (0.0)	4/30 (13.3)	3/16 (18.8)
24 to <60 months	4/4 (100.0)	1/1 (100.0)	22/40 (55.0)	9/10 (90)	4/5 (80)	34/41 (82.9)	11/12 (91.7)	6/7 (85.7)	24/30 (80.0)	12/16 (75.0)
Beginning & duration										
beginning (days after vaccination, mean±SD/N)	1.5±1/4	3±0/1	1.1±0.4/40	2±1.4/10	1.8±1.1/5	1±0.3/41	1.7±1.2/12	2.8±2.2/6	1.2±0.6/30	1.4±0.7/16
duration (days, mean±SD/N)	4.1±3.4/4	7±0/1	2.5±2.7/38	6.1±7/8	4.7±4.7/3	1.8±2.6/38	5.9±9/8	4.2±3.2/3	2.5±3/25	6.1±8/15

© 2022 Toepfner N et al. *JAMA Network Open.*

> 90 days	0/4 (0.0)	0/1 (0.0)	0/40 (0.0)	0/10 (0.0)	0/5 (0.0)	0/41 (0.0)	0/12 (0.0)	0/7 (0.0)	0/30 (0.0)	0/16 (0.0)
ongoing (days, mean±SD/N)	0±0/0	0±0/0	0±0/1	48.5±27.6/2	68±0/1	56±17/2	70.7±14.2/3	38±0/1	61.7±15.5/3	44±0/1
unknown	0/4 (0.0)	0/1 (0.0)	0/40 (0.0)	0/10 (0.0)	0/5 (0.0)	0/41 (0.0)	0/12 (0.0)	0/7 (0.0)	0/30 (0.0)	0/16 (0.0)
Disposition										
ambulatory	0/4 (0.0)	0/1 (0.0)	0/40 (0.0)	0/10 (0.0)	0/5 (0.0)	0/41 (0.0)	0/12 (0.0)	0/7 (0.0)	0/30 (0.0)	0/16 (0.0)
inpatient	0/4 (0.0)	0/1 (0.0)	0/40 (0.0)	0/10 (0.0)	1/5 (20.0)	0/41 (0.0)	0/12 (0.0)	1/7 (14.3)	1/30 (3.3)	0/16 (0.0)
mortality	0/4 (0.0)	0/1 (0.0)	0/40 (0.0)	0/10 (0.0)	0/5 (0.0)	0/41 (0.0)	0/12 (0.0)	0/7 (0.0)	0/30 (0.0)	0/16 (0.0)
other	0/4 (0.0)	0/1 (0.0)	3/40 (7.5)	0/10 (0.0)	0/5 (0.0)	1/41 (2.4)	1/12 (8.3)	0/7 (0.0)	1/30 (3.3)	0/16 (0.0)

Threat level from 0 (minimum) to 10 (maximum). Vac., vaccination. Ambulatory and inpatient refer to treatment requirement.

© 2022 Toepfner N et al. *JAMA Network Open.*

eTable 16. Dermatological symptoms

	local rash	rash all over the body	swelling of lymph nodes	painful lymph nodes	Extensive reddening of the skin	other skin dis-colora-tion	wheals/ hives	blisters with fluid /pus	Pe-techiae	Hema-toma	dry skin	skin ec-zema	open skin areas	It-ching	other skin com-plaints
After any vac.	78/242 (32.2)	13/242 (5.4)	69/242 (28.5)	20/242 (8.3)	5/242 (2.1)	1/242 (0.4)	14/242 (5.8)	7/242 (2.9)	11/242 (4.5)	3/242 (1.2)	37/242 (15.3)	21/242 (8.7)	4/242 (1.7)	27/242 (11.2)	8/242 (3.3)
After 1st vac.	46/242 (19.0)	7/242 (2.9)	29/242 (12.0)	7/242 (2.9)	3/242 (1.2)	1/242 (0.4)	3/242 (1.2)	5/242 (2.1)	8/242 (3.3)	2/242 (0.8)	20/242 (8.3)	10/242 (4.1)	3/242 (1.2)	16/242 (6.6)	6/242 (2.5)
After 2nd vac.	37/242 (15.3)	6/242 (2.5)	46/242 (19.0)	11/242 (4.5)	2/242 (0.8)	1/242 (0.4)	11/242 (4.5)	4/242 (1.7)	4/242 (1.7)	2/242 (0.8)	25/242 (10.3)	14/242 (5.8)	2/242 (0.8)	18/242 (7.4)	3/242 (1.2)
Threat level (mean±SD/N)	0.8±1.3/78	1.9±2/13	0.6±1/69	0.4±0.7/20	0.4±0.5/5	0±0/1	1.9±1.9/14	0.6±1.1/7	0.7±1/11	0.3±0.6/3	0.4±1/36	0.9±1.7/21	0±0/4	0.7±1.5/27	0.4±0.5/8
Dosage															
3µg	11/45 (24.4)	0/7 (0.0)	4/37 (10.8)	0/11 (0.0)	1/3 (33.3)	1/1 (100.0)	1/11 (9.1)	1/5 (20.0)	1/6 (16.7)	0/2 (0.0)	3/22 (13.6)	4/16 (25.0)	1/3 (33.3)	2/15 (13.3)	0/5 (0.0)
5µg	21/45 (46.7)	5/7 (71.4)	8/37 (21.6)	0/11 (0.0)	2/3 (66.7)	0/1 (0.0)	4/11 (36.4)	3/5 (60.0)	2/6 (33.3)	1/2 (50.0)	5/22 (22.7)	3/16 (18.8)	1/3 (33.3)	8/15 (53.3)	4/5 (80.0)
10µg	13/45 (28.9)	2/7 (28.6)	25/37 (67.6)	11/11 (100.0)	0/3 (0.0)	0/1 (0.0)	6/11 (54.5)	1/5 (20.0)	3/6 (50.0)	1/2 (50.0)	14/22 (63.6)	9/16 (56.3)	1/3 (33.3)	5/15 (33.3)	1/5 (20.0)
mixed/unknown	33/78 (42.3)	6/13 (46.2)	32/69 (46.4)	9/20 (45.0)	2/5 (40.0)	0/1 (0.0)	3/14 (21.4)	2/7 (28.6)	5/11 (45.5)	1/3 (33.3)	15/37 (40.5)	5/21 (23.8)	1/4 (25)	12/27 (44.4)	3/8 (37.5)
Age															
<12 months	3/78 (3.8)	0/13 (0.0)	0/69 (0.0)	0/20 (0.0)	0/5 (0.0)	0/1 (0.0)	0/14 (0.0)	1/7 (14.3)	0/11 (0.0)	0/3 (0.0)	0/37 (0.0)	1/21 (4.8)	0/4 (0.0)	0/27 (0.0)	0/8 (0.0)
12 to <24 months	23/78 (29.5)	3/13 (23.1)	10/69 (14.5)	0/20 (0.0)	0/5 (0.0)	1/1 (100.0)	2/14 (14.3)	2/7 (28.6)	1/11 (9.1)	1/3 (33.3)	5/37 (13.5)	4/21 (19.0)	2/4 (50.0)	7/27 (25.9)	0/8 (0.0)
24 to <60 months	52/78 (66.7)	10/13 (76.9)	59/69 (85.5)	20/20 (100.0)	5/5 (100.0)	0/1 (0.0)	12/14 (85.7)	4/7 (57.1)	10/11 (90.9)	2/3 (66.7)	32/37 (86.5)	16/21 (76.2)	2/4 (50.0)	20/27 (74.1)	8/8 (100)
Beginning & duration beginning (daysafter vaccination, mean±SD/N)	2.1±1.4/77	1.6±0.9/12	1.3±1/69	1.1±0.2/19	1.8±0.8/5	1±0/1	2.1±0.9/14	1.7±0.8/7	2.3±1.9/11	1.7±0.6/3	2.9±1.9/37	2.6±1.7/20	3±2.2/4	2.1±1.6/27	2.1±1.8/8
duration (days, mean±SD/N)	5.2±7.6/73	13.8±16.7/12	4.5±7.6/63	2.2±1.1/18	3.3±3/5	2±0/1	5.2±7.8/13	9±15.1/5	2.6±1.3/10	5±3.5/3	18.5±20.7/24	19.2±17.3/15	5.5±2.1/2	6.3±7.9/22	9.9±10.8/8

© 2022 Toepfner N et al. *JAMA Network Open.*

> 90 days	0/78 (0.0)	0/13 (0.0)	1/69 (1.4)	0/20 (0.0)	0/5 (0.0)	0/1 (0.0)	0/14 (0.0)	0/7 (0.0)	0/11 (0.0)	0/3 (0.0)	0/37 (0.0)	0/21 (0.0)	0/4 (0.0)	0/27 (0.0)	0/8 (0.0)
ongoing (days, mean±SD/N)	47±37.3/3	70±0/1	96±0/2	0±0/0	0±0/0	0±0/0	62±0/1	78.5±33.2/2	5±0/1	0±0/0	86.8±97.1/11	166.7±173.5/3	40±0/1	166.7±175.5/3	0±0/0
unknown	0/78 (0.0)	0/13 (0.0)	0/69 (0.0)	0/20 (0.0)	0/5 (0.0)	0/1 (0.0)	0/14 (0.0)	0/7 (0.0)	0/11 (0.0)	0/3 (0.0)	1/37 (2.7)	0/21 (0.0)	0/4 (0.0)	0/27 (0.0)	0/8 (0.0)
Disposition															
ambulatory	5/78 (6.4)	3/13 (23.1)	0/69 (0.0)	0/20 (0.0)	0/5 (0.0)	0/1 (0.0)	4/14 (28.6)	2/7 (28.6)	1/11 (9.1)	0/3 (0.0)	1/37 (2.7)	2/21 (9.5)	0/4 (0.0)	1/27 (3.7)	0/8 (0.0)
inpatient	0/78 (0.0)	0/13 (0.0)	0/69 (0.0)	0/20 (0.0)	0/5 (0.0)	0/1 (0.0)	0/14 (0.0)	0/7 (0.0)	0/11 (0.0)	0/3 (0.0)	0/37 (0.0)	0/21 (0.0)	0/4 (0.0)	0/27 (0.0)	0/8 (0.0)
mortality	0/78 (0.0)	0/13 (0.0)	0/69 (0.0)	0/20 (0.0)	0/5 (0.0)	0/1 (0.0)	0/14 (0.0)	0/7 (0.0)	0/11 (0.0)	0/3 (0.0)	0/37 (0.0)	0/21 (0.0)	0/4 (0.0)	0/27 (0.0)	0/8 (0.0)
other	1/78 (1.3)	1/13 (7.7)	0/69 (0.0)	0/20 (0.0)	0/5 (0.0)	0/1 (0.0)	0/14 (0.0)	0/7 (0.0)	0/11 (0.0)	0/3 (0.0)	2/37 (5.4)	0/21 (0.0)	0/4 (0.0)	1/27 (3.7)	1/8 (12.5)

Threat level from 0 (minimum) to 10 (maximum). Vac., vaccination. Ambulatory and inpatient refer to treatment requirement.

© 2022 Toepfner N et al. *JAMA Network Open.*

eTable 17. Non-BNT162b2 vaccines Since Jan. 15th 2022, n (%)

Vaccination	All<br>N=2945
Influenza	1355 (47.2)
Meningococcal	763 (26.6)
Measles/mumps/rubella with/without chickenpox	609 (21.2)
Tetanus/diphtheria/pertussis and/or pediatric polio	558 (19.4)
Hepatitis A/B	248 (8.6)
Human papillomavirus	6 (0.2)
Other	560 (19.5)

© 2022 Toepfner N et al. *JAMA Network Open.*

eTable 18. Counts of missing data, n/N(%)

	n=7806
Female	1 (0.0)
Male	1 (0.0)
Diverse	1 (0.0)
Age (years) (median, IQR)	3 (0.0)
Height (cm) (median, IQR)	108 (1.4)
Weight (kg) (median, IQR)	65 (0.8)
Comorbidities (yes)	0 (0.0)
Long-term Medication (yes)	22 (0.3)
Dosage	
Dosage 1st vaccination	566 (7.3)
Dosage 2nd vaccination	492/7102 (6.9)
Dosage 3rd vaccination	50/846 (5.9)
Symptoms	
Duration	106 (1.4)
Local	81 (1.0)
General	102 (1.3)
Fever	0 (0.0)
Musculoskeletal	120 (1.5)
Gastrointestinal	120 (1.5)
Otolaryngological	139 (1.8)
Pulmonary	142 (1.8)
Cardiovascular	139 (1.8)
Neurological	157 (2.0)
Psychological	157 (2.0)
Dermatological	171 (2.2)
Number absent days	333 (4.3)
Susceptibility to infections	176 (2.3)
Threat rating scale	266 (3.4)

© 2022 Toepfner N et al. *JAMA Network Open.*
